# Understanding the Relationship Between Official and Social Information About Infectious Disease: Experimental Analysis

**DOI:** 10.2196/25287

**Published:** 2021-11-23

**Authors:** Elias Assaf, Robert M Bond, Skyler J Cranmer, Eloise E Kaizar, Lauren Ratliff Santoro, Susumu Shikano, David J Sivakoff

**Affiliations:** 1 Pharmacy Analytics and Consulting Research Consulting Humana Louisville, KY United States; 2 The Ohio State University Columbus, OH United States; 3 Department of Communication The Ohio State University Columbus, OH United States; 4 Department of Political Science The Ohio State University Columbus, OH United States; 5 The University of Texas at Dallas Richardson, TX United States; 6 University of Konstanz Konstanz Germany

**Keywords:** disease, social information, official information, network experiments

## Abstract

**Background:**

Communicating official public health information about infectious diseases is complicated by the fact that individuals receive much of their information from their social contacts, either via interpersonal interaction or social media, which can be prone to bias and misconception.

**Objective:**

This study aims to evaluate the effect of public health campaigns and the effect of socially communicated health information on learning about diseases simultaneously. Although extant literature addresses the effect of one source of information (official or social) or the other, it has not addressed the simultaneous interaction of official information (OI) and social information (SI) in an experimental setting.

**Methods:**

We used a series of experiments that exposed participants to both OI and structured SI about the symptoms and spread of hepatitis C over a series of 10 rounds of computer-based interactions. Participants were randomly assigned to receive a high, low, or control intensity of OI and to receive accurate or inaccurate SI about the disease.

**Results:**

A total of 195 participants consented to participate in the study. Of these respondents, 186 had complete responses across all ten experimental rounds, which corresponds to a 4.6% (9/195) nonresponse rate. The OI high intensity treatment increases learning over the control condition for all symptom and contagion questions when individuals have lower levels of baseline knowledge (all *P* values ≤.04). The accurate SI condition increased learning across experimental rounds over the inaccurate condition (all *P* values ≤.01). We find limited evidence of an interaction between official and SI about infectious diseases.

**Conclusions:**

This project demonstrates that exposure to official public health information increases individuals’ knowledge of the spread and symptoms of a disease. Socially shared information also facilitates the learning of accurate and inaccurate information, though to a lesser extent than exposure to OI. Although the effect of OI persists, preliminary results suggest that it can be degraded by persistent contradictory SI over time.

## Introduction

During a contagious disease outbreak, public health campaigns provide people with relevant information, including symptoms and methods of transmission. The public’s understanding is critical for people to know which behaviors they should avoid and whether they should seek medical attention. To this end, public health campaigns are led by federal, state, local, and other organizations, which we refer to as *official* sources of information. However, many people may not directly encounter these campaigns and instead rely on information provided by social contacts, perhaps via social media. Rumors [1], lack of understanding [2], mis­ and disinformation campaigns [3], and motivated reasoning [4], among other factors, may inhibit understanding of the disease and contribute to a disconnect between accurate official information (OI) and inaccurate socially shared information. Furthermore, the spread of inaccurate information may have important downstream effects beyond the impact of the disease itself, such as on mental health [5].

Public health campaigns have been shown to be broadly effective. A recent examination of the COVID-19 pandemic in Italy found that most survey respondents knew of and believed in OI related to the disease [6]. This is particularly important, because in many ways socially shared misinformation about the disease was spreading more quickly than the disease itself [7]. In addition, experimental work demonstrates that individuals become more trusting of scientific experts and political actors with relevant expertise when encountering anxiety inducing external threats, such as H1N1 [8]. However, in addition to OI from the government and relevant policy actors, public health researchers have long recognized that people receive information from other sources, including media and friends, and that effective campaigns integrate these strategies [9]. A key challenge is understanding the impact that campaigns may have not only through direct exposure but also via social interactions and the sharing of disease-related information via social media.

People often rely on information from others to inform their own beliefs, attitudes, and behaviors related to health [10,11]. Studies have shown the importance of social networks in predicting health-related behaviors [12,13]. However, previous studies have not addressed the relative impact of socially transmitted information with respect to officially communicated public health information [see 14,15]. Specifically, van der Meer and Jin [14] studied the effectiveness of corrections to misinformation about infectious diseases coming from government health agencies, the news media, and social peers and found that government health agencies and the news media are more successful in combating misinformation about the disease than social peers. Similarly, Vraga and Bode [15] also looked at the effectiveness of corrections coming from the Centers for Disease Control and Prevention (CDC) or a social peer and found that the CDC is more effective in reducing misperceptions. Thus, engineers of public health interventions have little knowledge of how messages interact with socially spreading information. To address this, we used an experiment to causally test the hypothesis that health-related beliefs are socially influenced by the beliefs that others share with them in their social networks.

There is reason to believe that official, accurate health information is in competition with socially circulating inaccurate information. Rumors and misinformation are likely to spread across many domains [16]; research has shown this during recent infectious disease outbreaks, including HIV [17], H1N1 influenza [18], severe acute respiratory syndrome [19], the 2014-2015 Ebola outbreaks [20], the Zika epidemic [21], and the COVID-19 pandemic [7]. This information may be inaccurate and misinform the public or inhibit reliance on OI. Examining how these two information sources interact provides a deeper understanding of how the information environment leads to beliefs about an infectious disease.

This paper examines the interaction between official and social information (SI) about the disease. We use the term *social information* throughout, although we acknowledge that socially shared information differs from OI in many ways and that SI may be best understood as socially shared beliefs. To what extent does accurate, official public health information contribute to the learning of disease symptoms and transmission? To what extent does socially shared information magnify or inhibit learning? We used evidence from a randomized trial in a simulated network environment to shed light on these questions. In particular, we tested the hypotheses that participants who view relevant OI and those who view accurate SI will answer knowledge questions correctly more frequently than those with other informational experiences. We also examined whether these 2 effects reinforce each other. Finally, we tested the hypothesis that a positive teaching effect from a single viewing of OI can fade over time among those exposed to persistently inaccurate SI.

### Theory and Expectations

Individuals’ psychological dispositions interact with information from the external environment when making decisions [22]. However, individuals receive information from numerous sources. Individuals can receive information directly from the media, but most individuals are more likely to receive mediated information from opinion leaders in their social networks [23]. In today’s digital age, this *social communication* frequently comes via the internet, including social networking sites. On the internet, owing to the ease of digital publishing, everyone has the ability to become an opinion leader [24]. Individuals are then tasked with determining which information is correct or incorrect and whether and how to update their beliefs based on the information they consume.

Public health information from official sources, where official sources include public health agencies such as the CDC and the World Health Organization (WHO), is likely to be accepted as factual by the public [15]. This is primarily because the public views these sources as credible, conveying both trustworthiness and expertise [14]. Specifically, work in public health demonstrates that government health agencies and news media organizations are viewed as more credible and are more successful than social peers in increasing perceptions of the severity of a public health crisis [14].

Although research has consistently found that individuals accept and view information from official sources as credible, the reality that individuals receive much of their information from social sources makes the overall information environment more challenging to navigate. Information from the social environment can contain correct, factual information about a disease, such as repetition of information shared by the CDC or WHO, or it can be misleading or even completely false. During the 2018 Ebola outbreak, for example, inaccurate socially supplied information about the disease was widespread, and individuals who believed in the misinformation were less likely to adopt preventative behaviors such as agreeing to be vaccinated and seeking formal care [25]. Still, SI can also be helpful in correcting misinformation, though to a lesser extent than information shared from official sources [14,15,26]. Given the complex nature of the information environments that people encounter, it is critical to understand how people reconcile official and SI, especially when it conflicts.

Several other features of the information environments that people encounter may affect how they process the messages they encounter. First, communication environments, particularly social media, are often dynamic in that there may be repeated interactions between users over time, and the use and effects of social media can be reciprocal [27]. In such situations, the effects of exposure to initial messages may degrade or be reinforced through repeated or subsequent interactions [28]. Therefore, understanding how conflicting social and OI signals affect people’s attitudes over multiple interactions may provide a more nuanced understanding of how conflicting sources of information are processed.

In addition to being dynamic in time, communication on social media is often multidimensional even when it is limited to one overarching topic. For example, in the context of an infectious disease, two highly important dimensions of understanding the disease are the symptoms of the disease, so that a person may observe if they or others around them are likely to have contracted the disease, and how it spreads, so they know which actions are relatively risky or relatively safe in the context of the disease. Although it is important to know how people understand particular messages associated with either dimension in isolation, in many instances people process messages with multiple dimensions simultaneously, which may impact the effectiveness of a given message.

One of the primary features of social media is its ability to control self-presentation. Using the affordances of social media sites, users may selectively disclose information they wish their social contacts to know regarding their attitudes or beliefs [29] or may moderate their expressed attitudes because of their perception of the expectations of others [30]. In many instances, these processes are thought of in a research context as examples of social desirability bias, which are limitations of research designs or which research designs attempt to minimize to enable the observation of true attitudes or beliefs. Like many studies, our study cannot distinguish between sincerely and insincerely expressed beliefs. However, it is important to remember that even insincerely expressed attitudes or beliefs that are shared on social media platforms may still be taken at face value by others. Therefore, it is important to understand how attitudes and beliefs are expressed to others when people have the expectation that others will view those attitudes and beliefs. Because of the design of our study, we were able to examine the potential effects of the expression of beliefs, whether they were sincere or not.

Taken together, we expect that OI about a disease, that is, information received directly from official sources, will lead individuals to hold more factual beliefs about the disease. At the same time, socially shared information should lead to learning about disease, though to a lesser extent than OI. Specifically, we expect OI to be more effective in increasing learning about a disease when individuals are also exposed to accurate SI. On the other hand, we expect the effect of OI about a disease to persist but degrade as individuals are exposed to inaccurate SI over time.

## Methods

### Overview

To examine how official and SI interact in a dynamic information environment, we designed an experiment in which participants were exposed to both OI and structured SI over a series of ten rounds of computer-based interactions. The social connections between people are simulated with *bots* that are programmed to agree or disagree with key pieces of information in each round. These simulated alters allow us to control the social messaging received by each participant.

The research design for this study was reviewed and approved by the institutional review board of The Ohio State University (protocol #2014B0543).

### Participants

Participants fluent in German were recruited from a participant research pool of a European university in 2016 and were compensated with a modest monetary incentive. Students signed up to participate in short sessions scheduled during the course of a single week. All students participating in the same session were randomly assigned to the same treatment combination, as described below.

The experiment was conducted using the oTree [31] software. After providing consent, the participants began by answering demographic and opinion questions. Participants were then asked to read a description of the study, which explained that they would be asked questions and claimed that their answers would be shared with other participants (as described below, actual participant responses were not shared with other participants). Specifically, participants were told that they were embedded in a social network with other student participants contemporaneously completing the experiment, that their responses would be shared with other participants, and that they would view the responses of 3 other participants. However, no network was actually created, and participants viewed responses preprogrammed to mimic those observed in a previous set of experiments that involved actual networked interactions.

### Outcome Measures

Participants were then asked a battery of 14 true or false knowledge questions about hepatitis C, including six questions about the modes of transmission and eight about the symptoms. The topic of each question is listed in Table 1, and Multimedia Appendix 1 [32,33] contains the complete knowledge question text.

Our novel 14 question instrument gauges participants’ professed knowledge about hepatitis C. We reduce the impact of guessing by providing an uncertain answer, as well as true and false. The correct answers to all of the questions are presented in Table 1. Because participants learned about different topics related to hepatitis C at different times during the experiment (as described below), we did not combine participant responses across questions or across time points. Thus, we considered 14 separate outcome measures of professed knowledge, which correspond to indicators of a correct answer to each knowledge question. We did not distinguish between incorrect and uncertain responses in the analysis. Participant answers to the knowledge questions before exposure to treatments (as described in the *Design and Treatments* section below) provide a baseline to which we compare their future responses to the instrument as solicited throughout and at the end of the experiment.

**Table 1:** Summary of knowledge questions included in the experimental instrument, including the raw percent of participants who answered each question correctly at baseline, whether the question was subject to different social information, and the experimental round (if any) during which relevant official information was presented to participants in each intensity condition (high, low, and control). Note that infographics viewed by the low official information group were also viewed in the same round by participants in the high official information group.

Question topic	Correct answer	Correct at baseline (n=186), n (%)	Social manipulation	Round presented		
				High	Low	Control
Kissing	False	96 (51.6)	Different across conditions	1^a^	—^b^	—
Loss of appetite	True	81 (43.5)	Different across conditions	3^a^	3^c^	—
Headache	False	44 (23.7)	Different across conditions	—	—	—
Vomiting	True	78 (41.9)	Identical across conditions	3^a^	3^c^	—
Unprotected sex	True	154 (82.8)	Identical across conditions	5^a^	5^c^	—
Fever	True	127 (68.3)	Identical across conditions	7^a^	—	—
Fatigue	True	140 (75.3)	Identical across conditions	7^a^	—	—
Needle sharing	True	179 (96.2)	Identical across conditions	9^a^	9^c^	—
Breastfeeding	False	32 (17.2)	Identical across conditions	—	—	—
Diarrhea	False	35 (18.8)	Identical across conditions	—	—	—
Skin rash	False	43 (23.1)	Identical across conditions	—	—	—
Hair loss	False	83 (44.6)	Identical across conditions	—	—	—
Gym equipment	False	156 (83.9)	Identical across conditions	—	—	—
Tattoo equipment	True	165 (88.7)	Identical across conditions	—	—	—

^a^Official information treatment group.

^b^Not available.

^c^Social information treatment group.

We chose to measure knowledge about hepatitis C because it is a common disease with low public salience. Hepatitis C affects more than 3 million people in the United States (Department of Health and Human Services [34]), 14 million in Europe (WHO [35]), and approximately 71 million people worldwide (WHO [35]). We expected participants to have few entrenched beliefs and to find information about the disease novel. Summaries of the instrument responses reported in Table 1 show a range of baseline knowledge about hepatitis C topics, spanning from only 17.2% (32/186) reporting that hepatitis C is not spread via breastfeeding though an unsurprising 96.2% (179/186) reporting that hepatitis C is spread through sharing needles. The primary outcome measures capture changes in the proportion of correct answers within treatment groups, and the construction of these change score statistics is described in the *Statistical Analysis* section below.

### Design and Treatments

**Figure 1:**
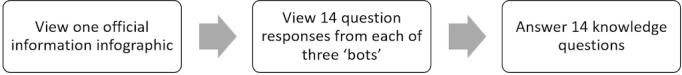
Each round of the experiment consists of three parts. First, participants are exposed to official information, then they are exposed to ostensibly social information, and finally they are asked to respond to 14 knowledge questions.

Ten experimental rounds were conducted at this point. We outline the overall structure of the rounds before describing how the treatments determine the nature of various components. Each round consisted of 3 parts, as shown in Figure 1. First, participants were exposed to an infographic intended to mimic information from a public health authority (*official* information). Some of this OI was related to knowledge questions about the modes of transmission and the symptoms of hepatitis C, and some were not. The infographics that were viewed in each round were determined by the treatment conditions, as described below (Table 1 for the infographic viewing schedule and question relevance and Multimedia Appendix 1 for the infographics themselves). Second, participants were exposed to ostensible SI from 3 other (purported) participants in the form of the participants’ most recent answers to the 14 knowledge questions. A depiction of this SI is provided in Multimedia Appendix 1. Third, participants were asked to answer the 14 knowledge questions.

We took care to create an environment in which participants believed that the observed SI was actually a response from other participants. First, participants completed the experiment in the same room as many other participants (sometimes participating in a different experiment). Second, our preprogrammed patterns of socially shared information were inspired by a pilot test of the experiment, such that relatively easy questions had more frequent accurate preprogrammed responses. Finally, we included time delays intended to mimic other participants that progressed more slowly. Although this SI does not mirror information shared from *friends*, it does correspond to information shared from casual connections across a social media platform.

Participants were assigned by session via cluster randomization to one of three degrees (*high, low,* or *control*) of relevant OI and to either reinforcing (*accurate*) or contradicting (*inaccurate*) SI conditions. After combining extremely small sessions (Multimedia Appendix 1), the 22 resulting sessions were assigned to the cross-classified conditions. Four sessions were assigned to accurate SI and high degree of relevant OI (total n=38), 4 sessions were assigned to accurate SI and low degree of relevant OI (total n=26), and 3 sessions were assigned to accurate SI and control for OI (total n=17). Four sessions were assigned to inaccurate SI and high degree of relevant OI (total n=33), 4 sessions were assigned to inaccurate SI and low degree of relevant OI (total n=33), and 3 sessions were assigned to inaccurate SI and control for OI (total n=39).

Depending on their assigned OI condition, each participant could view 5, 3, or 0 OI infographics, each of which contained information directly relevant to 1 or 2 of the knowledge questions during the course of the experiment. Table 1 identifies the questions for which the participants were assigned to the *high, low,* or *control* categories. OI treatment conditions were presented directly relevant OI and the round during which this OI was presented. Infographics that conveyed information about hepatitis C unrelated to contagion or symptoms were presented in the remaining 5-10 rounds. The infographics are shown in Multimedia Appendix 1. All presented OI infographics contain accurate information.

**Figure 2:**
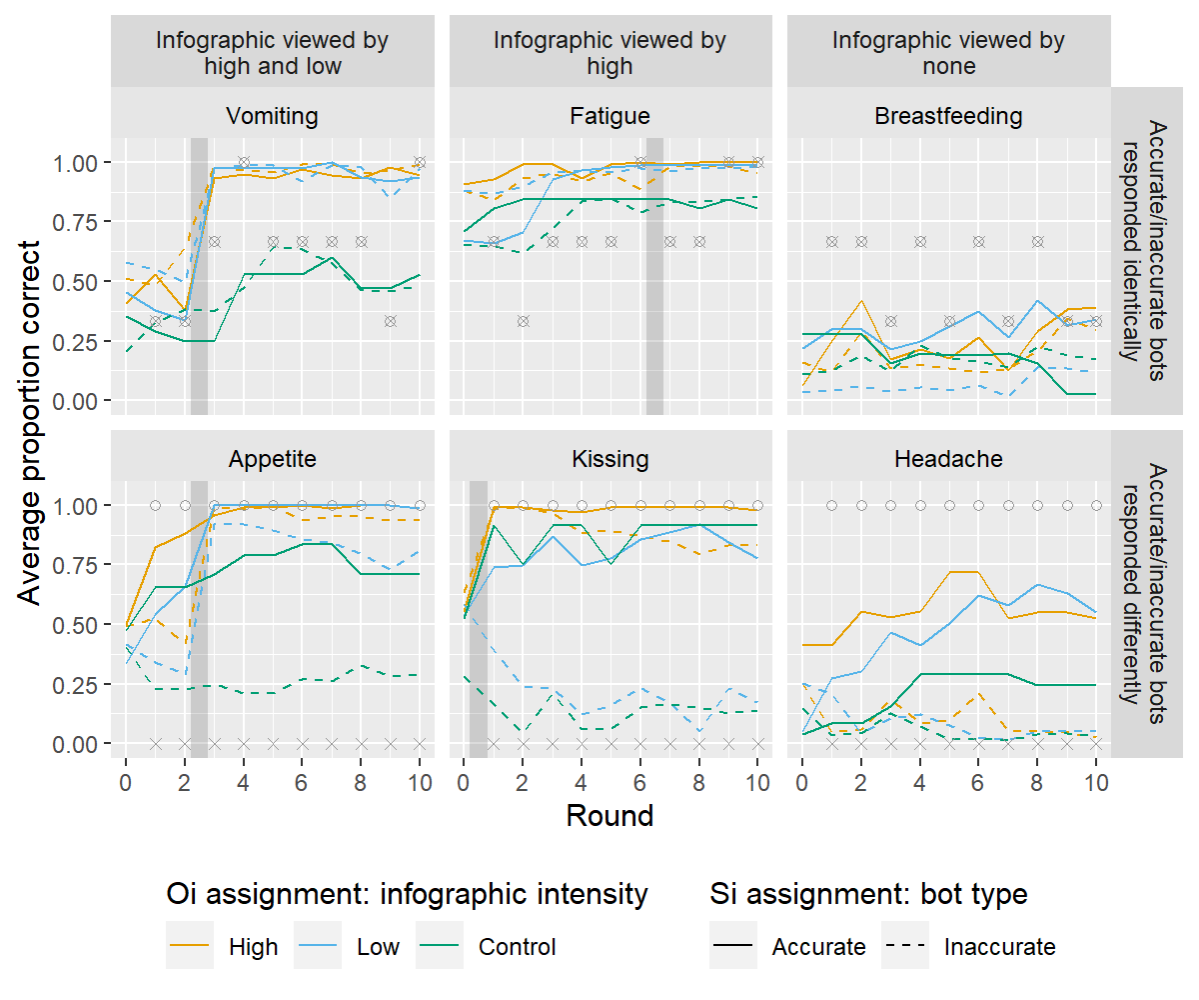
Proportion of correct responses for a representative subset of knowledge questions, averaged across sessions within the same cross-classified treatment assignments, as indicated by line color (official information [OI]) and type (social information [SI]). Direct interplay with treatment assignments differs across questions, as indicated by the columns (OI) and rows (SI) of panels. For OI, relevant infographics are viewed by the groups of participants indicated in the column heading in the round indicated by the vertical gray bar. For SI, the proportion of correct answers provided by bots are displayed as o and the proportion of incorrect answers provided by bots are displayed as x symbols. These symbols are always overlaid in the first row.

The SI treatment conditions were intended to provide some participants with systematically accurate social influence and others with systematically inaccurate social influence, while maintaining the plausibility of real response sharing among participants. Thus, all participants viewed identical sequences of pre­programmed SI responses for 11 of the 14 knowledge questions. These pre­programmed responses were designed to mimic real participant responses to the same battery of questions deployed in a similar experiment. For the three remaining questions related to kissing, loss of appetite, and headache, participants assigned to the *accurate* SI condition were led to believe the three friends responded with false, true, and false (respectively, to the three questions) across all ten rounds, which are the correct responses. Those assigned to the *inaccurate* SI condition were led to believe the 3 friends responded with true, false, and true (respectively, to the 3 questions) across all ten rounds, which are the incorrect responses. Summaries of the displayed SI (bot) responses for 6 of the questions are depicted as circles and crosses in Figure 2. Multimedia Appendix 1 provides more information about the treatments and random assignment. At the end of each session, participants were provided with all correct factual information and were debriefed.

### Statistical Analysis

For exploratory data analyses, we present question-specific longitudinal trends in the average proportion of correct responses across sessions stratified by treatment assignment. We colloquially refer to increases in the percentage of responses that are correct as learning, but recognize that these changes could also reflect changes in expressed attitudes rather than sincerely held beliefs. For formal statistical tests, we capture learning across rounds separately for each knowledge question via differences in average log odds ratios across sessions stratified by treatment assignment. This approach is similar to a random effects logistic regression where round is treated as a categorical factor but allows us to implement adjustments (Multimedia Appendix 1), which makes our calculations computationally feasible where classical random effects logistic regression is not.

Differences in learning over rounds and across treatment groups were summarized by subtracting the appropriate average log odds or log odds ratios. Most within-group summary statistics compared the log odds of a correct answer in a particular round to those at baseline, and some compared responses at round ten with those in the round where correct OI was viewed.

Finally, we compare learning across groups by taking the differences in within-group average log odds ratios across relevant treatment conditions. Large differences across treatment conditions imply that treatment causes differences in learning. We used permutation to approximate the exact reference distributions. To test the equality of learning across groups, we estimated 2-sided *P* values with the proportion of summary measures from 5000 permuted assignments that were further from zero than the summary measure based on the true assignment. Permutation-based tests rely on minimal assumptions but also typically have relatively low statistical power. Additional details regarding the statistical analyses are presented in Multimedia Appendix 1. A significance level of *P*=.05 was used for all tests. All analyses were conducted using R statistical software [32].

## Results

### Overview

A total of 195 participants across 23 sessions consented to participate; only data for the 186 who completed all ten rounds were included in the primary analyses and are described in Table 2. As noted in the table, 65.1% (121/186) of respondents were female, and their average age was 23 years. Overall, 56.5% (105/186) of respondents reported attending university for 4 or fewer semesters. Further demographic summaries as well as examination of missing data patterns and randomization balance are included in the Multimedia Appendix 1.

We tested four expectations linked to the relationship between accurate official public health information and accurate SI. These expectations imply that increases in correct responses across rounds (which we term *learning*) will differ across the 6 treatment groups in various ways for various questions.

**Table 2:** Baseline demographic and other characteristics for study completers (n=186).

Characteristic		Values
Age (years), mean (SD; range)		22.85 (2.44; 19 to 31)
Sex (female), n (%)		121 (65.1)
**Parental socioeconomic status, self-assessed (n=180), n (%)**		
	Limited	35 (19.4)
	Middle	95 (52.8)
	Upper	50 (27.8)
**Childhood environment, self-assessed (n=185), n (%)**		
	City	47 (25.4)
	Small town	61 (32.9)
	Suburbs	11 (5.9)
	Country	66 (35.7)
Freshmen or sophomore class rank, n (%)		105 (56.5)
Political science major, n (%)		34 (18.3)
Political orientation score^a^ (n=159), mean (SD; range)		−0.38 (0.75; −2 to 2)

^a^Political orientation was self-assessed on a 5-point scale from *very left* to *very right* and then recoded as integers from −2 to +2. Note that our European university recruitment pool provided little racial and ethnic diversity.

### Learning From OI

W**e** expect viewing OI to increase the learning of information about a disease, as previous literature demonstrates [6,8,15]. Those assigned to the high, low, and control intensity OI groups saw infographics relevant to seven, four, and zero questions. Thus, for 7 questions, we expect those who saw a relevant infographic (ie, those in higher intensity groups) to have greater learning for that question as compared with those who did not.

Visualization of data summaries highlights trends that may support our expectations. The lines in Figure 2 depict trends in the proportion of correct responses for each of the 6 groups (where color indicates OI treatment assignment and line type indicates SI treatment assignment) for 6 representative questions. Because all bots gave the same responses for the questions in the first row (as indicated by superimposed *x* and *o* symbols), these questions straightforwardly address the effect of OI. The leftmost plot in this row summarizes the responses to questions related to vomiting. The gray-shaded vertical bar indicates that a relevant infographic was shown to groups assigned to high-and low-intensity treatments before they answered the knowledge questions in round 3. For these 2 groups (yellow and blue lines), we see a dramatic increase in the proportion correct between rounds 2 and 3, suggesting that these groups learned from the relevant infographics. In contrast, we do not see similar learning among those assigned to the control intensity condition (green lines), suggesting that these groups did not learn from the irrelevant infographics they were shown.

For the question related to fatigue, we expected to see a similarly large learning in round 7 among the high intensity groups (and not among the low and control intensity groups who did not view a relevant infographic). However, differences in participant answers did not clearly follow this pattern, perhaps because of high baseline knowledge about this symptom or consistent longitudinal trends in bot responses. Further experiments are required to confirm this hypothesis. Finally, as expected, we see no substantial sustained learning about the potential contagion of breastfeeding in any group, as no group was shown a relevant infographic. Among the questions in the second row where the SI treatments differed, we also see substantial learning in rounds where relevant infographics are viewed, although these trends are complicated by differential socially shared information, as explained below.

**Figure 3:**
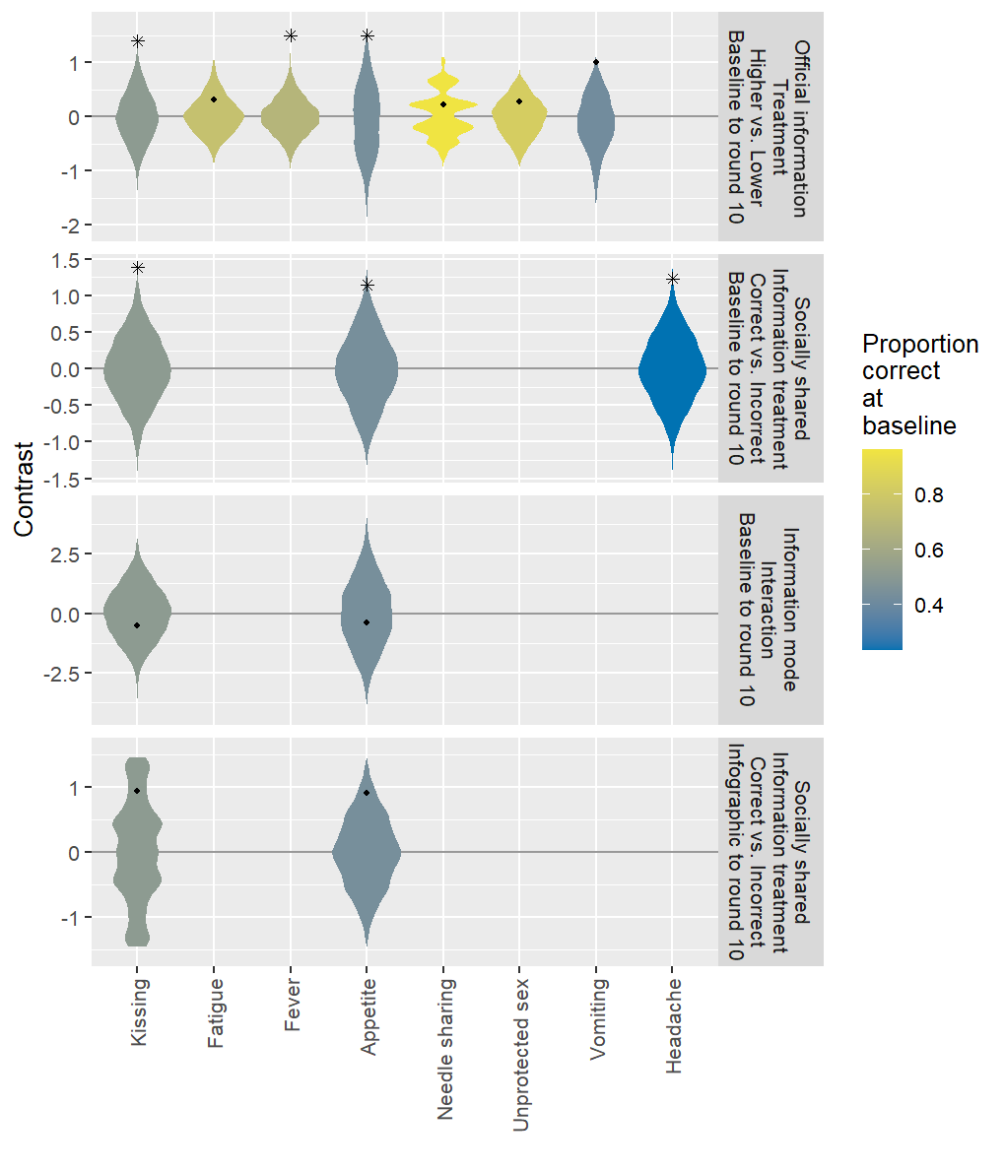
Observed (points) and permutation reference distributions (violins) for observed contrasts in average log odds of correct responses across treatment assignment groups (first two rows) and in difference-in-difference of average log odds (third row). Stars indicate permutation *P*≤.05.

Formal tests that compare groups assigned to view relevant infographics with those that did not are summarized for germane questions in the first row of Figure 3, where stars indicate statistically significant differences in the average log odds of a correct question response across groups defined only by the OI treatment assignment. We found statistically significant differences in learning from baseline to round ten for all questions where the baseline knowledge was at most moderate (less than 75% correct), except for the question related to vomiting (*P*=.051). The results of pairwise comparisons across the assigned information treatment groups for all questions are included in Multimedia Appendix 1.

### Learning From Socially Shared Information

We expect (in) accurate socially shared information to affect learning of (in) correct information. For three of the questions (related to kissing, appetite, and headache), the (in) accurate bot responses were always (in) accurate; for the remaining 11 questions, all responses were identical across both types. Thus, for the 3 questions, we expect those assigned to the accurate bot treatment groups to learn more than those assigned to the inaccurate bot groups. The second row of Figure 2 shows the response trends for these three questions, where the differential bot responses are indicated by 100% (3/3) and 0% (0/0) accurate responses for the accurate (*o*) and inaccurate (x) bots, respectively. Because no group was shown an infographic directly relevant to headache, this question (rightmost column) directly addresses the effect of SI. We see that across the ten rounds, those groups with whom accurate information is socially shared (solid lines) have positive learning, whereas those socially receiving inaccurate information (dashed lines) have flat or negative learning. In other questions, we see similar trends among groups that do not view relevant infographics (ie, the low and control intensity groups for the kissing question and the control group for the appetite question). The second row of Figure 3 shows that permutation tests confirm strong statistically

significant differences in learning from baseline to round 10 across the assigned bot types for all three questions, where the information shared by bots differed (all *P≤*.01). Note that the participants had poor-to-moderate baseline knowledge for all three questions.

### Interaction Between Information Sharing Modes

To the extent that official and SI interact, we expect OI to overwhelm SI. That is, we expect the effect of socially shared information to be smaller within groups who viewed germane infographics as compared with those who did not view germane infographics. Infographics relevant to appetite and kissing were shown to some groups, and the accurate and inaccurate bots differed in their responses to these questions. Thus, we focus on these two questions to explore information-mode interactions.

**Figure 4:**
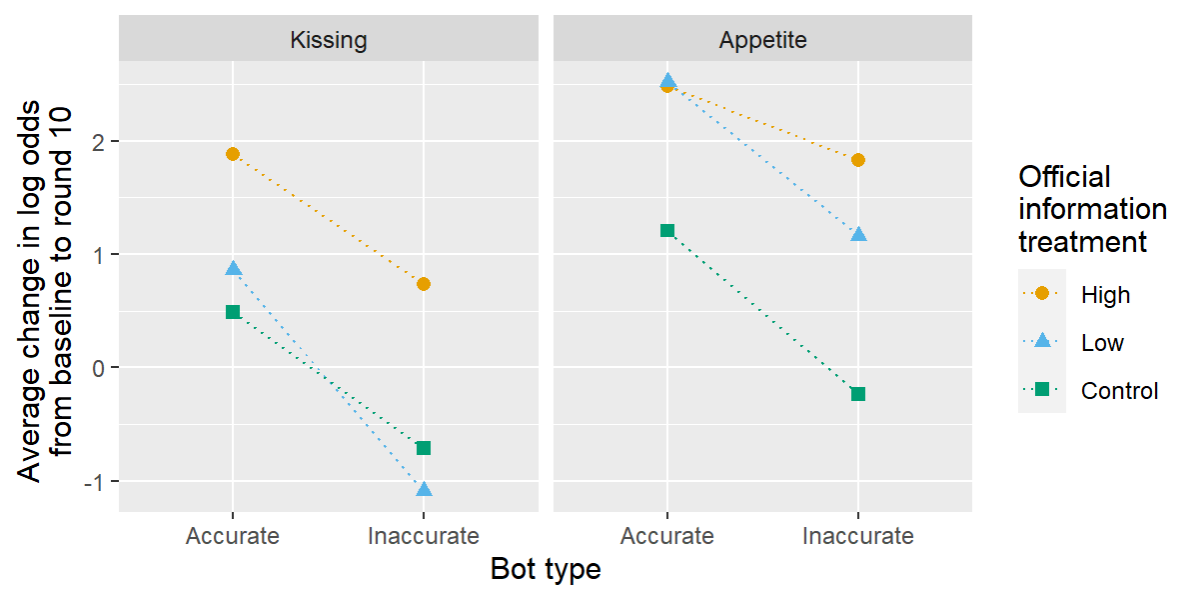
Treatment group-specific change in average log odds from baseline to round ten. Dotted lines connect pairs of groups with the same official information treatment group; parallel lines suggest no interaction between information modes.

First, we consider the question related to appetite (lower left panel of Figure 2). Because all groups had similar knowledge at baseline, the effect of socially shared information can be approximated by the group differences in the average proportion correct at round 10. The effect of socially shared information is approximated by the difference between the corresponding solid and dashed lines. These differences are relatively small for the groups that viewed germane infographics (yellow and blue) and much larger for the groups that did not view germane infographics (green lines). This pattern is also present for the kissing question and seems to suggest that the effect of socially shared information is greater for groups that did not view germane infographics. However, this perception may be influenced by the boundary effects of the proportion scale. Figure 4 suggests that such differences disappeared after changing to the log odds scale. The log odds-based permutation tests (third row of Figure 3) confirm no strong evidence of an interaction between the modes of information sharing.

### Persistence of OI

Although we saw no global interactions among the effects of mode on learning from baseline to round 10, we explored a fourth, more targeted hypothesis that the effect of receiving official public health information persists but degrades in the face of contradictory SI. For the question related to appetite, we see that high- and low-information intensity groups achieved nearly unanimously correct responses after viewing the infographics in round 3 (all 4 yellow and blue lines in Figure 2). Of these, groups that are also assigned to socially receive accurate information (yellow and blue solid lines) retain a nearly unanimously correct response proportion at the end of the experiment. However, those groups with which inaccurate information is socially shared (yellow and blue dashed lines) tend to have small declines in the proportion of correct answers by round ten. We see similar trends for the high information intensity group (yellow lines) in answering the question related to kissing. The bottom row of Figure 3 confirms that these trends may be unusual relative to permuted treatment assignments, but the strength of evidence does not reach statistical significance (*P*=.29 and *P*=.09 for kissing and appetite, respectively).

It may also appear that there is more degradation in learning because of inaccurate SI for participants in the low OI group versus the high OI group (Figure 4, right panel, blue vs orange), and it is possible that participants receiving less OI overall might be more susceptible to social influence. However, we have only 2 groups with which to measure the variability of the persistence of learning because of OI; so, we did not attempt a formal comparison between these groups.

## Discussion

### Principal Findings

This work demonstrates that both official and SI influence people’s reported understanding of infectious diseases. As expected from the previous literature, exposure to official public health information about hepatitis C increased the learning related to its spread and symptoms. Learning followed socially supplied information, whether accurate or inaccurate, though to a lesser extent. Trends in our data suggest that learning from OI is remarkably resilient even in the presence of persistent contradictory SI, although some modest degradation may be hidden by low statistical power. In an era in which official public health campaigns are frequently in competition with information shared on social media, this study provides some reason to be optimistic that public health campaigns may be able to overcome socially shared misperceptions.

Our work is consistent with previous work on misperceptions in the context of health, which finds that corrective information may inhibit false beliefs [14,15,36]. A web-based experiment investigating corrections to misinformation about a disease outbreak found that official sources of information were more effective at correcting misinformation than peers [14]. Our work examines the competing relationship between official and SI, rather than corrections to misinformation. However, we find that OI sources are much more effective at inducing changes in expressed beliefs, which is consistent with this previous work.

This study addresses the longitudinal effect of SI about diseases in tandem with official public health information in an experimental setting. Many studies of this type would include a one-shot intervention or follow up with participants after a short period. Here, we investigated how expressed beliefs update over multiple rounds and through multiple interactions with members of their experimentally constructed social networks. Because of this, we are able to provide a more nuanced understanding of competing messages in dynamic information environments.

It is important to note that our study used expressed beliefs about hepatitis C as the primary dependent variable. Of course, nearly any study of beliefs about disease relies on expressed beliefs in one way or another. However, because our study is premised on participants’ understanding that they are viewing the responses of other participants who are completing the study contemporaneously, these expressed beliefs may be impacted by not only their own beliefs and any changes in them because of the experimental stimuli but also by social desirability bias that may impact their expressed beliefs [37]. Studies on the expression of belief in political falsehoods [38] have shown that the strategic expression of insincerely expressed beliefs is modest. However, the knowledge that you are sharing beliefs, whether sincerely held or not, means that these are beliefs that the participant expected others to see and experience. In this way, this work explores whether sincerely held beliefs are affected by official sources of information or not and whether the information environment becomes more clearly aligned with public health officials, reducing the degree of conflict between official and SI sources.

Although our work focuses on the context of a contagious disease, the results may contribute to our understanding of how social media may affect other aspects of health or may relate to other domains. Of course, OI may be in competition with (or be reinforced by) socially shared information across a number of domains, including other diseases, such as Zika [15], or aspects of health such as mental health [39]. Future work may wish to investigate whether and how socially circulating information affects beliefs in OI across other domains.

### Strengths and Limitations

Our ability to control available information lends strong credibility to the causal interpretation of our results. Experiments on networks help bridge the gap between observational studies of people in their natural social environments and lab experiments that abstract away social influences.

Reliable extension of our conclusions to real-world situations relies on participants’ interpretation of the information presented as legitimate. This hurdle may be easier to clear for OI than for SI. One major strength of our controlled experiment is that participants were embedded in an environment that promoted realism in a fictional social network.

This experiment has several important limitations, which we elaborate on here. First, the social networks we examined appeared to be among participants who were anonymous to one another within the setting of the experiment. Although this situation may be encountered on an anonymous message board or comment thread, it is possible that in real social networks, in which participants have strong social bonds and reputations, the effects of SI may be different. Future work may wish to investigate whether and how SI is transmitted in social networks among participants who are already connected to one another.

Second, our experiment limited SI responses to true, false, or unsure. In reality, people make intentional and nuanced attempts to convince others in the social sphere of their beliefs. It is difficult to envision a controlled experiment in which participants are allowed to communicate so freely and also maintains a degree of control that enables clear causal interpretation, but this should be addressed in future studies. We view this as a trade-off between tight control that enables us to make clear causal claims and less tight control that not only enables a greater variety of communication but also reduces the ability to interpret the social component in a clear causal fashion.

Third, the participants in our study were all similar in age. Previous work in a different context (politics) found that an official message with a reinforcing social component was more effective at changing behavior among older participants than an official message without the social component [40]. It is possible that the limited interaction we find between social and OI owes to the age distribution of the participants of the study, the fact that the official and SI are contradictory, to the fact that our study examines health information, or to some other factor. Future work may examine contradicting official and SI in a more diverse sample to understand whether heterogeneous treatment effects exist.

Fourth, the study did not test for potential mediating and moderating variables that may explain how or to what degree official and SI affect beliefs. This limitation is related to, but distinct from, concerns about how anonymous networks may impact network effects. Mediating and moderating relationships may be a result of aspects of existing relationships, in which case anonymous studies would help to control for these effects but would also limit researchers’ ability to examine them. However, it is also possible that mediating or moderating relationships are a result of aspects of relationships between people that develop rapidly, perhaps during the course of a few interactions. In this case, even in a short network experiment among anonymous individuals, mediating or moderating relationships may be examined. For example, the degree of trust in information sources may affect the degree to which participants respond to the beliefs shared by either official or SI sources. In this study, participants were informed that they would be connected to other anonymous participants; so, any interpersonal trust would have to be established during the course of the study. We believe it is unlikely that, in the context of this study, varying levels of trust would be created during the course of the study. However, in real-world situations in which people have ongoing relationships with those from whom they receive SI, trust and reputation are likely to impact how people process and respond to the messages shared by their social contacts. Future studies may wish to investigate trust, perhaps through experiments that enable researcher control over not only message content but also cues related to trust. We note that trustworthiness is one of multiple possible mediating or moderating variables that may enable a more nuanced understanding of the competing or reinforcing effects of social and OI.

Finally, the small number of experimental sessions limit the power of permutation-based statistical inference. The permutation-based approach used here makes very few assumptions and, therefore, enables us to make clear claims based on the statistical evidence that does not depend on common assumptions used in model-based analyses that may not hold for the data we collected. Future reanalyses based on longitudinal models may provide more nuanced, though more model-dependent results. In addition, reliance on college student volunteers and participant buy in to treatment legitimacy raises external validity concerns.

Nonetheless, our results provide some good news for sponsors of public health campaigns even in a time of overwhelming prevalence of SI with a wide range of accuracy. Consistent fact-based official messages can break through the SI noise.

### Conclusions and Future Work

This experiment is one of multiple related projects examining social processes of information spread in networks and includes 2 additional experiments that address some of these limitations. First, human participants are randomly assigned to prespecified networks, which provides insight into how aspects of the network structure affect the diffusion of information about the disease. Second, participants share disease information with individuals in a real-world social network, providing a test of the external validity of our findings. Although this analysis focuses solely on the *bots* experiment, future work will incorporate the other 2 experiments. Although the studies of actual networks will enable the analysis of more complex network relationships, they will have a substantial trade-off in the degree of researcher control over what participants experience.

In the real world, our results present a puzzle for web-based health communities, particularly those that are moderated by professionals. In such communities, moderators may play a role similar to that of the official sources of information in our experiment [41,42]. In such communities, it may be possible for moderators to leverage network effects to broaden the impact of their official message. Web-based health communities often provide opportunities for users to connect in anonymous ways and must develop relationships with other users over time, similar to the design of our experiment. However, it is important to note that in such communities, professionals and moderators may have an outsized influence and the positive impact that they may have can lead to the propagation of misinformation just as it may lead to the spread of correct information [43,44]. Future work may wish to further investigate the interaction of official and SI or instances in which the line between them is not as clear, such as when a moderator or user of a web-based health community presents themselves as having expertise and other users must evaluate the user’s credibility.

Our results underscore the importance of public health practitioners taking into account the effects of both OI sources, which may have a degree of control over, and SI sources, over which they may have limited control. We also show that OI is substantially more effective at promoting learning than socially supplied information, underscoring the importance of public health campaigns for inducing correct beliefs. Although we do not find evidence that official and SI interact, it is possible that the effects of such an interaction are small and nuanced, and we would nonetheless encourage practitioners to consider how OI may be transmitted through social ties.

## Appendix

Multimedia Appendix 1 Supplementary information concerning the design, analysis, results, examples of the treatments used, and primary dependent variable measurement.

## Abbreviations

CDC: Centers for Disease Control and Prevention
OI: official information
SI: social information
WHO: World Health Organization
