# Supplementary material for: Understanding the Relationship Between Official and Social Information About Infectious Disease: Experimental Analysis
Source: J Med Internet Res. 2021 Nov 23;23(11):e25287. doi: 10.2196/25287 (PMC8663576; doi:10.2196/25287)
Supplement: Multimedia Appendix 1 [file jmir_v23i11e25287_app1.docx]

# Contents

**Supplementary Appendices**

1. [Design 1](#_TOC_250001)
   1. Treatment Conditions and Assignment 1
      1. Oﬀicial Information (OI) Treatment 2
      2. Social Information (SI) Treatment 2
      3. Treatment Assignment 2
   2. [Summary Measures and Statistical Analyses 3](#_TOC_250000)
2. Results 5
   1. Sample, Missing Data and Balance 5
      1. Missing Data 5
      2. Randomization Balance 5
   2. Longitudinal Trends 9
   3. Tests of Hypotheses 12
3. Social Information Communications 22
4. Oﬀicial Information Posters 23
5. Symptom and Contagion Questions 29

References 30

# A Design

Participants were recruited as a part of a set of similar experiments, some of which required a fixed number of participants. Some or all volunteers at sessions with more or fewer participants than needed for the fixed number experiment were invited to participate in the ‘bots’ experiment described and analyzed here. Participants were blinded to the exact experiment in which they participated. Participants sat at computers in a lab with dividers to prevent participants from casually viewing each other’s screens. After providing consent, the participants began by answering a number of demographic and opinion questions. Participants were then asked to read a description of the study, which explained that they would be asked questions and falsely claimed that their answers would be shared with other participants. (We designed the experiment to lend plausibility to this false claim by placing participants in a room with many people appearing to be simultaneously participating in the same experiment.) The study explanation also noted that participants may not view the same information throughout the experiment. Participants were then asked a battery of 14 knowledge questions about Hepatitis C, including 6 questions about modes of transmission and 8 about symptoms (see Figure A.1). At this point, the ten experimental rounds commenced. During each round, participants

1. viewed information formatted to resemble a poster that could be posted in the community (e.g., on a bus), which we term ’oﬀicial information,’ or OI,
2. viewed purported responses from three other participants, which we term ’friend responses’, and
3. answered the battery of 14 knowledge questions.

At the end of the ten rounds, participants were provided with correct answers to the 14 knowledge questions, as extracted from the Center for Disease Control and Prevention (CDC) website.

## Treatment Conditions and Assignment

Within this general framework, we implemented two experimental treatments: the oﬀicial information (OI) treatment and the social information (SI) treatment.

#### Oﬀicial Information (OI) Treatment

Depending on their assigned OI condition, each participant could view from zero to five OI ‘posters’ that contain information directly relevant to one or more of the knowledge questions. Table A.1 identifies the questions for which participants assigned to the ‘high’, ‘low’, or ‘control’ OI treatment conditions were presented directly relevant OI, and the round during which this OI was presented. Posters that conveyed information about Hepatitis C unrelated to contagion or symptoms were presented in the remaining five to ten rounds. The posters are shown in Appendix D. All presented OI posters contained accurate information.

#### Social Information (SI) Treatment

The social information treatment conditions were intended to provide some participants with systematically correct social influence and others with systematically incorrect social influence, while maintaining the plausibility of real response sharing among participants. Thus, all participants viewed identical sequences of pre-programmed friend responses for eleven of the fourteen knowledge questions. These pre-programmed responses were designed to mimic real observed participant responses to the same battery of questions deployed in a similar experiment. For the three remaining questions related to kissing, loss of appetite and headache, participants assigned to the ‘correct’ SI condition were led to believe all three friends responded with False, True and False (respectively to the three questions) across all ten rounds, which are the correct responses. Those assigned to the ‘incorrect’ SI condition were led to believe all three friends responded with True, False and True (respectively to the three questions) across all ten rounds, which are the incorrect responses. Summaries of the displayed friend responses are depicted as circles and crosses in Figure 2 in the main document and Figure B.1 in these Appendices. An image of how social information was viewed by participants during the questionnaire stage of each round is in Figure C.1.

#### Round Presented

**at Baseline**

| **Question Topic** | **Correct Answer** | **Percent Correct** | **Social Manipulation** | **High** | **Low** | **Control** |  |
| --- | --- | --- | --- | --- | --- | --- | --- |
| Kissing | False | 51.6 | Different Across Conditions | 1 | – | – |  |
| Loss of Appetite | True | 43.5 | Different Across Conditions | 3 | 3 | – |  |
| Headache | False | 23.7 | Different Across Conditions | – | – | – |  |
| Vomiting | True | 41.9 | Identical Across Conditions | 3 | 3 | – |  |
| Unprotected Sex | True | 82.8 | Identical Across Conditions | 5 | 5 | – |  |
| Fever | True | 68.3 | Identical Across Conditions | 7 | – | – |  |
| Fatigue | True | 75.3 | Identical Across Conditions | 7 | – | – |  |
| Needle Sharing | True | 96.2 | Identical Across Conditions | 9 | 9 | – |  |
| Breastfeeding | False | 17.2 | Identical Across Conditions | – | – | – |  |
| Diarrhea | False | 18.8 | Identical Across Conditions | – | – | – |  |
| Skin Rash | False | 23.1 | Identical Across Conditions | – | – | – |  |
| Hair Loss | False | 44.6 | Identical Across Conditions | – | – | – |  |
| Gym Equipment | False | 83.9 | Identical Across Conditions | – | – | – |  |
| Tattoo Equipment | True | 88.7 | Identical Across Conditions | – | – | – |  |

Table A.1: Knowledge question summary, including the raw percent of participants who answered the question correctly at baseline, whether they were subject to different social information, and the experimental round (if any) during which relevant information was presented to participants in each intensity condition (high, low and control).

#### Treatment Assignment

To simplify the implementation, each session was randomly assigned to a single cross-classification of treatment conditions in a block-randomized manner. After collapsing extremely small sessions (see Section B.1.1 below), the 22 sessions were assigned to the cross-classified conditions as shown in Table A.2. The total number of participants assigned to each cross-classification, as well as the minimum and maximum number of participants per session, are also shown in the table.

#### Information Intensity Social Manipulation High Low Control

Correct 4 (38; 8-12) 4 (26; 4-10) 3 (17; 3-8)

Incorrect 4 (33; 5-11) 4 (33; 5-13) 3 (39; 9-15)

Table A.2: Number of sessions assigned to each cross-classified pair of conditions. Numbers in parentheses are the total number of participants across all sessions and the range of numbers of participants per session

## A.2 Summary Measures and Statistical Analyses

Preliminary data analyses to assess the missing data pattern and randomization balance were conducted via standard tests of means, probabilities and log odds that rely on exact or asymptotic distributions. Fisher’s exact test and Mantel-Haenszel tests are examples of the former; likelihood ratio tests in meta-regression are examples of the latter. The application of each test is described in more detail in Section B.1.1.

In the main results, we focus on a single summary measure: the session- and question-specific knowledge improvement from some round to a later round. For most analyses, we focus on this improvement from baseline to round 10. We visualize trends with round-specific averages of session-specific proportions, weighted by the inverse session-specific variance of the proportion (estimated with a 0.5 continuity correction to ensure finite weights). For tests, we reduce boundary effects by using log odds transformations of session-specific proportions (employing a 0.5 continuity correction for all questions and sessions to ensure that all transformations are finite).

We compare conditions as follows. For each relevant condition, collapsed conditions (e.g., combined High and Low oﬀicial information for questions where both of these groups viewed a relevant infographic) or cross-classification of conditions, we compute a single summary statistic by taking the average of the session-specific difference in log odds ratio (i.e., the difference in log odds for the desired round (e.g., round 10) versus baseline), weighted by the inverse estimated session-specific variance of the log odds ratio. We then compare conditions by creating a test statistic that is simply the difference between relevant summary statistics. We construct reference distributions by permuting the condition(s) assigned to each session. For comparisons of conditions in only one treatment (i.e., only information intensity or only social information type), these permutations are stratified by the treatment not involved in the comparisons. For comparisons involving both treatments, we permute the cross-classified assignment to maintain the joint distribution of assigned conditions. In each case, we employ 5000 permutations.

All analyses were conducted using R statistical software (R Core Team, 2019). Meta-analytic analyses were completed using the metafor package (Viechtbauer, 2010).

Sind die folgenden Aktivitäten Übertragungswege von Hepatitis C?

*Are the following ways that you might contract Hepatitis C?*

#### Glauben Sie, dass Hepatitis C übertragen werden kann durch:

***Do you believe that you can contract Hepatitis C from:***

schweißige Turn- und Fitnessgeräte? *Sweaty gym equipment?*

das Küssen einer infizierten Person? *Kissing an infected person?*

das Stillen eines Kindes? *Breastfeeding?*

ungeschützten Geschlechtsverkehr mit einer in- fizierten Person?

ein Tattoo-Equipment, das an einer infizierten Per- son benutzt wurde?

*Unprotected sex with an infected person?*

*Tattoo equipment used on an infected per- son?*

das Teilen von Injektionsnadeln? *Needle sharing?*

Sind die folgenden Symptome, mögliche Symptome von Hepatitis C?

*Are the following Symptoms of Hapatitis C?*

**Hepatitis-C-Symptom? *Symptom of Hepatitis C?***

Hautausschlag *Rash*

Kopfweh *Headache*

Erbrechen *Vomiting*

Fieber *Fever*

Erschöpfung *Fatigue*

Appetitlosigkeit *Loss of Appetite*

Figure A.1: Knowledge questions presented to participants in German. English translations are provided for reader convenience. Possible responses are ”Ja”, ”Nein” and ”Unsicher” (”Yes”, ”No” and ”Unsure”).

# Results

## Sample, Missing Data and Balance

There were 195 participants. 186 of these had complete responses for all ten rounds of the experiment, which corresponds to a 4.6% non-response rate. The primary analyses only include those participants who fully completed all ten rounds, as the non-response rate is small and we saw no substantial evidence against a missing completely at random missing data mechanism.

#### Missing Data

The experiments included a small amount of participant drop out, where we did not record responses for all knowledge questions across all ten rounds. We examined possible deviations from a completely at random missing data pattern via three types of tests. First, we tested whether participants across treatment condition assignments display differential rates of drop-out. Table B.1 presents P values from Fisher’s exact tests of independence between treatment assignment and drop-out, where we excluded from the analyses any session that experienced no drop-out, but did not further stratify by session. Second, we tested whether baseline knowledge for those who dropped out differed from those who fully completed all ten rounds. Table B.2 presents P values from Mantel-Haenszel tests of the independence of baseline correct response and completion status. For each test, we condition on session. Note that sessions without any drop-out do not contribute to Mantel-Haenszel test statistics. Third, we tested whether a selection of demographic variables and other characteristics differed for those who dropped out. These variables were chosen a priori to include standard adjustment characteristics and other variables thought to be both relevant to the research questions at hand and having enough variation to be statistically relevant. Table B.3 summarizes the results. For continuous variables, the summary is the estimated mean for completers minus that for those who dropped out, and the P value is calculated based on a random-effects meta-analysis that assumes constant variance across missing data groups within each session, and independent participant values across sessions. The constant within-session variance assumption allows us to estimate the difference in means even for sessions where only one participant dropped out. For binary variables, the summary is the estimated common session-specific odds ratio, where values greater than one indicate positive association between completing all ten rounds and the indicator variable as described in the table. The binary variable P values are calculated via exact Mantel-Haenszel tests stratified by session (where sessions without any dropout do not contribute to the analysis). For categorical variables with three or more levels, we also report P values from exact Mantel-Haenszel tests, but do not report any single number summary. The large P values in these results indicate little evidence against a completely random missing data mechanism. Two exceptions are for age, where the average completer age is about two years older than those who dropped out, and for government response, where everyone who did not complete the study does not believe government is responding well to Hepatitis C. On the whole, we are confident that proceeding with a complete case analysis will have little impact on the validity of our results.

| Treatment | *P* value |
| --- | --- |
| Oﬀicial Information Treatment | 0.22 |
| Social Information Treatment | 0.14 |

Table B.1: P values for Fisher’s Exact tests of independence between treatment assignment and drop-out indicator, unconditional on the session.

After accounting for missing responses, there was one session that included only one participant and a second session that included only two. To balance privacy concerns and analytic ease with accuracy, we combine these two exceedingly small sessions to create a single pseudo-session with three participants, which for convenience we will continue to refer to as a ‘session.’

#### Randomization Balance

We examined the balance across treatment condition groups. First, we examined the differences in baseline knowledge across the conditions within each treatment. Table B.4 summarizes tests for association between

Summary *P* Value

| Question Topic  Kissing | 1.65 0.70 | |
| --- | --- | --- |
| Fever | 3.97 | 0.37 |
| Fatigue | Inf | 0.10 |
| Vomiting | 1.96 | 0.44 |
| Loss of Appetite | 7.58 | 0.08 |
| Unprotected Sex | 2.16 | 0.67 |
| Needle Sharing | 0.62 | 0.54 |
| Breastfeeding | 0.73 | 1.00 |
| Diarrhea | 0.00 | 0.59 |
| Skin Rash | 0.69 | 1.00 |
| Headache | 0.61 | 1.00 |
| Hair Loss | 0.78 | 1.00 |
| Gym Equipment | 0.84 | 1.00 |
| Tattoo Equipment | 0.58 | 0.61 |

Tattpp Equipment

Table B.2: *P* values for Mantel-Haenszel tests of independence of baseline correct response and drop-out indicator, conditional on the session.

condition for a single treatment factor and log odds of a correct answer to the knowledge question at baseline. *P* values are based on tests from random-effects logistic meta-regression of the session-specific counts of correct responses to each question on treatment condition indicators, and assumes participant responses in different sessions to be independent. We see from this table that there is little evidence of imbalance in any baseline knowledge across the social information condition groups. However, we do see evidence of imbalance across the oﬀicial information treatment condition groups, particularly for the questions related to fatigue, vomiting, headache, and gym equipment. Examination of Figures in the main text and Figure B.1 gives insight as to the ordering of this knowledge, where sessions assigned to the high OI intensity group tend to be more knowledgeable at baseline and the sessions assigned to the control OI intensity group tend to be less knowledgeable at baseline. To mitigate this pattern, our main analyses rely only on outcomes in any round relative to the response at an earlier round (e.g., baseline).

Second, we examined differences in demographic variables and other participant characteristics across treatment conditions. Table B.5 summarizes the results from random effects meta-regressions of estimates on treatment condition indicators, where participant outcomes are considered independent across sessions and possibly correlated within sessions. For continuous variables, the reported summary is the estimated variable mean within each condition stratum and the *P* value is from the accompanying omnibus test from random effects meta-regressions of the covariate on treatment condition indicators. For binary variables, the reported summaries are log odds ratios from similar logistic meta-regressions, as well as their accompanying test results. For multi-category variables, we do not report single-number summaries, and base tests on multivariate baseline category logistic meta-regression. Based on these results, we see little evidence of demographic or participant characteristic imbalance across treatment condition groups.

| Covariate | Type | Summary | *P* Value |
| --- | --- | --- | --- |
| Age | Continuous | 2.17 | 0.01 |
| Female Gender | Binary | 0.16 | 0.11 |
| Considers themselves to be a religious person | Binary | 1.02 | 1.00 |
| Self-described parental class (lower, middle, upper) | Multi-category | - | 0.89 |
| Type of formative area (city, suburbs, town, country) | Multi-category | - | 0.22 |
| Has 10 or more close friends | Binary | 1.16 | 1.00 |
| Is close to 15 or more family members | Binary | 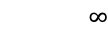   \|  \| \| --- \| | 0.59 |
| Talks to close family members weekly or more frequently | Binary | 0.49 | 0.47 |
| Has attended university for four or fewer semesters | Binary | 0.78 | 1.00 |
| Major related to politics or management | Binary | 0.29 | 0.08 |
| Political Orientation (5-level scale, where -2= "very left" and +2 = "very right" | Continuous | 0.16 | 0.65 |
| Believes the government/health agencies are responding well to Hepatitis C | Binary | 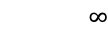 | 0.01 |

Table B.3: Summaries and *P* values for tests of differences in demographic and other characteristics for those who dropped out versus those who completed all 10 rounds. For continuous variables, the summary is the estimated mean for completers minus that for those who dropped out, and the *P* value comes from a random-effects meta-analysis that assumes constant variance across missing data groups within sessions and independence across sessions.

#### Balance *P* Value

**Correct at Baseline**

| **Question** | **OI Condition** | **SI Different** | **Marginal Proportion** | **OI** | **SI** |  |
| --- | --- | --- | --- | --- | --- | --- |
| Kissing | High | Yes | 0.52 | 0.09 | 0.56 |  |
| Fatigue | High | No | 0.75 | 0.04 | 0.71 |  |
| Fever | High | No | 0.68 | 0.07 | 0.31 |  |
| Loss of Appetite | Low | Yes | 0.44 | 0.45 | 0.93 |  |
| Needle Sharing | Low | No | 0.96 | 0.79 | 0.99 |  |
| Unprotected Sex | Low | No | 0.83 | 0.09 | 0.71 |  |
| Vomiting | Low | No | 0.42 | 0.01 | 0.99 |  |
| Headache | None | Yes | 0.24 | 0.01 | 0.81 |  |
| Tattoo Equipment | None | No | 0.89 | 0.15 | 0.91 |  |
| Gym Equipment | None | No | 0.84 | 0.05 | 0.71 |  |
| Hair Loss | None | No | 0.45 | 0.26 | 0.88 |  |
| Skin Rash | None | No | 0.23 | 0.11 | 0.43 |  |
| Diarrhea | None | No | 0.19 | 0.17 | 0.16 |  |
| Breastfeeding | None | No | 0.17 | 0.99 | 0.23 |  |

Table B.4: *P* values for tests of independence of baseline correct response and treatment condition. *P* values are derived from random-effects meta-regressions.

#### OI Condition SI Condition Covariate Type High Low Control *P* Value Correct Incorrect *P* Value

Age Continuous 22.74 22.63 23.32 0.42 22.68 22.97 0.47

Female Gender Binary 0.59 0.72 0.67 0.49 0.65 0.67 0.85

Considers themselves to be a re- ligious person

Self-described parental class (lower, middle, upper)

Type of formative area (city, sub- urbs, town, country)

Binary 0.38 0.31 0.41 0.49 0.36 0.37 0.85

Multi-category – – – 0.55 – – 0.14

Multi-category – – – 0.64 – – 0.18

Has 10 or more close friends Binary 0.09 0.10 0.13 0.77 0.09 0.12 0.53

Is close to 15 or more family members

Talks to close family members weekly or more frequently

Has attended university for four or fewer semesters

Major related to politics or man- agement

Political Orientation (5-level scale, where -2 = ”very left” and

+2 = ”very right”)

Believes the government/health agencies are responding well to Hepatitis C

Binary 0.11 0.12 0.14 0.87 0.09 0.15 0.20

Binary 0.61 0.45 0.61 0.16 0.56 0.55 0.89

Binary 0.52 0.59 0.60 0.71 0.57 0.56 0.91

Binary 0.18 0.15 0.21 0.70 0.15 0.21 0.30

Continuous -0.57 -0.26 -0.54 0.05 -0.46 -0.46 1.00

Binary 0.49 0.53 0.53 0.86 0.53 0.49 0.62

Table B.5: *P* values for tests of association between demographic or other participant characteristics and treatment condition based on random effects meta-regression. Reported summaries are estimated group-specific means for continuous variables and probabilities for binary variables. *P* values are those associated with the omnibus test for inclusion of the treatment in the regression model.

8

## Longitudinal Trends

Figure B.1 shows the longitudinal trend in the session-average proportion correct across the ten rounds for each of the eight knowledge questions not presented in the main text, as well as the rounds in which relevant OI was presented and a summary of the friend responses presented at each round. Figure B.2 presents the longitudinal trends in the log odds of a correct answer relative to baseline for each of the eight knowledge questions not presented in the main text.

9

Figure B.1: Proportion of correct responses for the knowledge questions not presented in the main figures, averaged across sessions within the same cross-classified treatment assignments, as indicated by line color and type. Condition attributes are depicted by shaded stripes (times between question rounds when oﬀicial information is viewed by session participants assigned to certain information intensity conditions) and symbols (proportion of friends represented as having the correct answer to session participants assigned to certain social information conditions). OI=Oﬀicial Information; SI=Social Information.

10

11

Figure B.2: Difference in log odds of a correct response at a given round versus at baseline for the knowledge questions not presented in the main figures, averaged across sessions within the same cross-classified treatment assignments, as indicated by line color (OI) and line type (SI). Condition attributes are depicted by shaded stripes (times between question rounds when oﬀicial information is viewed by session participants assigned to certain information intensity conditions) and symbols (proportion of friends represented as having the correct answer to session participants assigned to certain social information conditions). OI=Oﬀicial Information; SI=Social Information.

## Tests of Hypotheses

We formally test four sets of hypotheses. First, we consider the effect of OI on the log odds of a correct response. Figures B.3 and B.4 depict the permutation reference distributions (violins) and observed values (dots) for the difference in relative knowledge improvement across OI conditions. This summary statistic is akin to the coeﬀicient in a logistic regression for the interaction between an indicator of round (ten or baseline) and an indicator of OI condition. As noted in the main text, for questions with relevant OI presented only to the high intensity OI condition (orange), we expect to see large differences for comparisons between the high and each of the low and control OI conditions (the left and center violins in each panel). For questions with relevant OI presented to the high and low intensity OI conditions (blue), we expect to see large differences for comparisons between each of the high and low intensity OI conditions and the control condition (the center and right violins in each panel). For questions with no relevant OI presented to any participants, we expect to see no large differences. Table B.6 summarizes the results of pairwise comparisons across assigned information treatment groups.

Figure B.3: Permutation-based reference distributions (violins) and observed values (dots) comparing relative knowledge improvement across groups defined by information intensity assignment for a representative subset of knowledge questions. Colors indicate which questions were affected by various oﬀicial information conditions.

Second, we assess the effect of social information on the log odds of a correct response. Figures B.5 and B.6 depict the permutation reference distributions for the difference in relative knowledge improvement across SI conditions. This summary statistic can be thought of as the coeﬀicient in a logistic regression for the interaction between an indicator of round (10 or baseline) and an indicator of SI condition. As noted in the

**Difference in Log Odds Ratios (*P* Value)**

| **Question Topic** | **Baseline Correct** | **Oﬀicial Info.** | **Social Info.** | **High vs. Control** | **High vs. Low** | **Low vs.**  **Control** | **Correct**  **Incorrect** |  |
| --- | --- | --- | --- | --- | --- | --- | --- | --- |
| Kissing | 0.52 | High | Different | 1.52 (**< .01**) | 1.31 (**0.01**) | 0.22 (0.70) | 1.38 (**< .01**) |  |
| Fatigue | 0.75 | High | Identical | 0.41 (**0.33**) | 0.02 (**0.95**) | 0.39 (0.36) | 0.25 (0.44) |  |
| Fever | 0.68 | High | Identical | 1.65 (**< .01**) | 1.35 (**< .01**) | 0.30 (0.48) | 0.21 (0.48) |  |
| Loss of Appetite | 0.44 | High & Low | Different | 1.84 (**0.01**) | 0.52 (0.45) | 1.32 (**0.06**) | 1.15 (**0.01**) |  |
| Needle Sharing | 0.96 | High & Low | Identical | 0.18 (**0.72**) | -0.09 (0.84) | 0.27 (**0.58**) | 0.18 (0.68) |  |
| Unprotected Sex | 0.83 | High & Low | Identical | 0.36 (**0.37**) | 0.13 (0.75) | 0.24 (**0.57**) | 0.58 (0.08) |  |
| Vomiting | 0.42 | High & Low | Identical | 1.17 (**0.04**) | 0.37 (0.52) | 0.80 (**0.16**) | 0.52 (0.08) |  |
| Headache | 0.24 | None | Different | -0.31 (0.50) | -0.52 (0.23) | 0.21 (0.66) | 1.23 (**< .01**) |  |
| Tattoo Equipment | 0.89 | None | Identical | 0.40 (0.48) | 0.68 (0.18) | -0.28 (0.64) | 0.55 (0.21) |  |
| Gym Equipment | 0.84 | None | Identical | 0.04 (0.93) | -0.11 (0.81) | 0.16 (0.76) | 0.25 (0.55) |  |
| Hair Loss | 0.45 | None | Identical | -0.23 (0.68) | -0.34 (0.50) | 0.12 (0.83) | -0.39 (0.39) |  |
| Skin Rash | 0.23 | None | Identical | 0.01 (0.98) | -0.93 (0.02) | 0.94 (0.03) | -0.62 (0.06) |  |
| Diarrhea | 0.19 | None | Identical | -1.53 (< .01) | -0.56 (0.31) | -0.97 (0.08) | -0.89 (0.03) |  |
| Breastfeeding | 0.17 | None | Identical | 0.89 (0.04) | 0.32 (0.44) | 0.57 (0.20) | 0.01 (0.96) |  |

**vs.**

Table B.6: Permutation-based *P* values comparing relative knowledge improvement across information intensity groups and across social information groups. Bold-face indicates *P* values hypothesized to be small. Baseline Correct is the proportion of participants who had a correct response at baseline, which indicates the diﬀiculty of the question. Info. = Information.

main text, we expect to see large differences for questions where the presented friend responses differed across SI condition (blue), and not for those where the presented friend responses were identical across conditions (orange).

Third, we considered the interaction between the OI and SI conditions in overall relative knowledge improvement. While we hypothesized that the OI effect is larger than the SI effect, our data did not provide strong evidence to support this hypothesis. Tables B.7 and B.8 show *P* values for the effects of oﬀicial and social information, respectively, within strata defined by the other treatment factor. Table B.9 contains permutation-based *P* values for the interactions, or differences in differences. We speculate that our experiment does not have suﬀicient power to reveal such patterns without adopting stronger modeling assumptions.

Fourth, we examined the ‘staying power’ of the OI effect in the face of contradictory social information. Figures B.7 and B.8 depict the permutation distribution for and observed round-specific log odds of a participant correctly responding for those questions where directly relevant OI was presented, stratified by the assigned OI condition. The leftmost column depicts the log odds at baseline, the center column depicts the log odds at the end of the round in which the question-relevant OI was presented, and the rightmost column depicts the log odds at the end of round ten. For all questions and OI condition assignments (all rows), we expect and largely see no difference across SI condition groups (no difference between dots within the same panel) at baseline. For questions with identical friend responses across SI conditions (orange), we also expect to see no difference between SI condition groups at the relevant OI round or round ten. For questions with different friend responses across SI conditions (blue), we expect to see a small difference across SI condition groups at the relevant OI round, as the OI effect is expected to strongly pushes nearly all responses to be correct, but a larger difference at round ten, as the effect of OI may degrade as participants forget the OI but the influence of SI persists. Some instances where we see unexpected differences may in part be due to very high rates of correct responses. Table 2 in the main text includes corresponding *P* values that tests for relative differences between the SI conditions across the OI round and round ten.

Figure B.4: Permutation-based reference distributions (violins) and observed values (dots) comparing relative knowledge improvement across groups defined by information intensity assignment for the knowledge questions not presented in the main figures. Colors indicate which groups received directly relevant official information.

Figure B.5: Permutation-based reference distributions (violins) and observed values (dots) comparing relative knowledge improvement across groups defined by social information assignment for a representative subset of knowledge questions. Colors indicate which questions were part of the social information treatment.

Figure B.6: Permutation-based reference distributions (violins) and observed values (dots) comparing relative knowledge improvement across groups defined by social information assignment for a representative subset of knowledge questions. Colors indicate which questions were part of the social information treatment.

17

-0.38 (0.71)

| **Stratum** | | | | | | | |
| --- | --- | --- | --- | --- | --- | --- | --- |
| **Question Topic** | **Baseline Correct** | **Question OI** | **Social Information** | **Contrast** | **Correct** | **Incorrect** |  |
| Kissing | 0.52 | High | Different | HighVsControl | 1.39 (**0.17**) | 1.45 (**0.13**) |  |
|  |  |  |  | HighVsLow LowVsControl | 1.02 (**0.26**)  0.37 (0.73) | 1.83 (**0.03**) |  |

Fatigue 0.75 High Identical HighVsControl 1.22 (**0.06**) -0.05 (**0.93**)

HighVsLow 0.24 (**0.70**) 0.08 (**0.89**)

LowVsControl 0.98 (0.15) -0.13 (0.84)

| Fever | 0.68 | High | Identical | HighVsControl | 1.84 (**0.02**) | 1.41 (**0.06**) |
| --- | --- | --- | --- | --- | --- | --- |
|  |  |  |  | HighVsLow LowVsControl | 1.29 (**0.05**)  0.55 (0.43) | 1.29 (**0.05**) |
| Loss of Appetite | 0.44 | High and Low | Different | HighVsControl | 1.28 (**0.28**) | 2.07 (**0.06**) |
|  |  |  |  | HighVsLow LowVsControl | -0.04 (0.98)  1.31 (**0.26**) | 0.67 (0.56) |
| Needle Sharing | 0.96 | High and Low | Identical | HighVsControl | 0.86 (**0.17**) | -0.58 (**0.34**) |
|  |  |  |  | HighVsLow LowVsControl | 0.86 (0.17)  0.00 (**1.00**) | -0.98 (0.11) |
| Unprotected Sex | 0.83 | High and Low | Identical | HighVsControl | 0.34 (**0.56**) | -0.16 (**0.79**) |
|  |  |  |  | HighVsLow LowVsControl | 0.69 (0.20)  -0.35 (**0.57**) | -0.46 (0.41) |
| Vomiting | 0.42 | High and Low | Identical | HighVsControl | 1.55 (**0.06**) | 0.98 (**0.23**) |
|  |  |  |  | HighVsLow LowVsControl | 0.23 (0.78)  1.32 (**0.10**) | 0.52 (0.51) |
| Headache | 0.24 | None | Different | HighVsControl | -0.86 (0.34) | -0.73 (0.42) |
|  |  |  |  | HighVsLow LowVsControl | -1.06 (0.20)  0.20 (0.82) | -0.75 (0.35) |
| Tattoo Equipment | 0.89 | None | Identical | HighVsControl | 0.72 (0.27) | -0.13 (0.84) |
|  |  |  |  | HighVsLow LowVsControl | 0.67 (0.32)  0.05 (0.92) | 0.28 (0.68) |
| Gym Equipment | 0.84 | None | Identical | HighVsControl | 0.80 (0.28) | -0.41 (0.58) |
|  |  |  |  | HighVsLow LowVsControl | -0.18 (0.80)  0.98 (0.17) | 0.22 (0.75) |
| Hair Loss | 0.45 | None | Identical | HighVsControl | 0.46 (0.53) | -0.37 (0.62) |
|  |  |  |  | HighVsLow LowVsControl | -0.92 (0.17)  1.38 (0.05) | 0.13 (0.85) |
| Skin Rash | 0.23 | None | Identical | HighVsControl | 0.55 (0.41) | 0.05 (0.94) |
|  |  |  |  | HighVsLow LowVsControl | -0.74 (0.23)  1.30 (0.04) | -0.93 (0.12) |
| Diarrhea | 0.19 | None | Identical | HighVsControl | -0.28 (0.74) | -2.09 (0.01) |
|  |  |  |  | HighVsLow LowVsControl | -0.45 (0.59)  0.17 (0.84) | -0.58 (0.48) |
| Breastfeeding | 0.17 | None | Identical | HighVsControl | 1.89 (< .01) | 0.25 (0.71) |
|  |  |  |  | HighVsLow | 0.37 (0.53) | 0.23 (0.71) |
|  |  |  |  | LowVsControl | 1.52 (0.02) | 0.01 (0.99) |

0.12 (0.85)

1.40 (**0.22**)

0.39 (**0.58**)

0.30 (**0.62**)

0.46 (**0.60**)

0.02 (0.98)

-0.41 (0.58)

-0.63 (0.40)

-0.49 (0.50)

0.98 (0.13)

-1.51 (0.07)

Table B.7: Permutation-based *P* values for the effect of the oﬀicial information treatment (the difference in relative knowledge improvement across oﬀicial information groups) within social information groups.

18

|  | | | | | **Stratum** |  |  |
| --- | --- | --- | --- | --- | --- | --- | --- |
| **Question Topic** | **Baseline Correct** | **Question OI** | **Social Information** | **High** | **Low** | **Control** |  |
| Kissing | 0.52 | High | Different | 1.14 (**0.21**) | 1.95 (**0.02**) | 1.20 (**0.26**) |  |
| Fatigue | 0.75 | High | Identical | 1.00 (0.11) | 0.84 (0.18) | -0.28 (0.70) |  |
| Fever | 0.68 | High | Identical | 0.21 (0.72) | 0.21 (0.71) | -0.22 (0.76) |  |
| Loss of Appetite | 0.44 | High and Low | Different | 0.65 (**0.58**) | 1.36 (**0.20**) | 1.44 (**0.24**) |  |
| Needle Sharing | 0.96 | High and Low | Identical | 1.35 (0.01) | -0.48 (0.45) | -0.09 (0.82) |  |
| Unprotected Sex | 0.83 | High and Low | Identical | 1.20 (0.02) | 0.05 (0.93) | 0.70 (0.26) |  |
| Vomiting | 0.42 | High and Low | Identical | 0.01 (0.99) | 0.30 (0.70) | -0.56 (0.54) |  |
| Headache | 0.24 | None | Different | 1.44 (**0.08**) | 1.76 (**0.04**) | 1.57 (**0.13**) |  |
| Tattoo Equipment | 0.89 | None | Identical | 0.95 (0.14) | 0.56 (0.40) | 0.09 (0.85) |  |
| Gym Equipment | 0.84 | None | Identical | 0.65 (0.34) | 1.04 (0.10) | -0.56 (0.49) |  |
| Hair Loss | 0.45 | None | Identical | -0.47 (0.49) | 0.57 (0.40) | -1.30 (0.08) |  |
| Skin Rash | 0.23 | None | Identical | -0.35 (0.57) | -0.54 (0.41) | -0.85 (0.23) |  |
| Diarrhea | 0.19 | None | Identical | -0.15 (0.86) | -0.27 (0.74) | -1.95 (0.02) |  |
| Breastfeeding | 0.17 | None | Identical | 0.28 (0.63) | 0.15 (0.79) | -1.36 (0.06) |  |

Table B.8: Permutation-based *P* values for the effect of the social information treatment (the difference in relative knowledge improvement across SI groups) within oﬀicial information groups.

19

|  | | | | | **Value (P-Value)** |  |  |
| --- | --- | --- | --- | --- | --- | --- | --- |
| **Question** | **Baseline Correct** | **Info** | **Social Information** | **High vs. Control** | **High vs. Low** | **Low vs. Control** |  |
| Kissing | 0.52 | High | Different Across Conditions | -0.06 (0.96) | -0.81 (0.54) | 0.75 (0.61) |  |
| Fatigue | 0.75 | High | Identical Across Conditions | 1.28 (0.17) | 0.16 (0.85) | 1.11 (0.24) |  |
| Fever | 0.68 | High | Identical Across Conditions | 0.44 (0.66) | 0.00 (1.00) | 0.43 (0.67) |  |
| Loss of Appetite | 0.44 | Low | Different Across Conditions | -0.79 (0.64) | -0.70 (0.65) | -0.09 (0.96) |  |
| Needle Sharing | 0.96 | Low | Identical Across Conditions | 1.44 (0.10) | 1.83 (0.02) | -0.39 (0.65) |  |
| Unprotected Sex | 0.83 | Low | Identical Across Conditions | 0.50 (0.55) | 1.15 (0.13) | -0.65 (0.44) |  |
| Vomiting | 0.42 | Low | Identical Across Conditions | 0.57 (0.64) | -0.29 (0.79) | 0.86 (0.47) |  |
| Headache | 0.24 | None | Different Across Conditions | -0.13 (0.93) | -0.31 (0.79) | 0.19 (0.89) |  |
| Tattoo Equipment | 0.89 | None | Identical Across Conditions | 0.85 (0.36) | 0.39 (0.66) | 0.47 (0.61) |  |
| Gym Equipment | 0.84 | None | Identical Across Conditions | 1.21 (0.23) | -0.39 (0.68) | 1.61 (0.11) |  |
| Hair Loss | 0.45 | None | Identical Across Conditions | 0.83 (0.44) | -1.05 (0.27) | 1.88 (0.05) |  |
| Skin Rash | 0.23 | None | Identical Across Conditions | 0.50 (0.59) | 0.19 (0.84) | 0.31 (0.74) |  |
| Diarrhea | 0.19 | None | Identical Across Conditions | 1.80 (0.13) | 0.12 (0.91) | 1.68 (0.15) |  |
| Breastfeeding | 0.17 | None | Identical Across Conditions | 1.64 (0.08) | 0.13 (0.88) | 1.51 (0.11) |  |

Table B.9: Permutation-based *P* values comparing the effect of the SI treatment (the difference in relative knowledge improvement across SI groups) across information intensity groups.

Figure B.7: Permutation-based reference distributions (violins) and observed values (dots) comparing relative knowledge across groups defined by social information assignment for a representative subset of knowledge questions. The analyses are stratified by oﬀicial information assignment and displayed only for those strata that were shown relevant oﬀicial information. Colors indicate which questions were part of the social information treatment.

Figure B.8: Permutation-based reference distributions (violins) and observed values (dots) comparing relative knowledge across groups defined by social information assignment for a representative subset of knowledge questions. The analyses are stratified by oﬀicial information assignment and displayed only for those strata that were shown relevant oﬀicial information. Colors indicate which questions were part of the social information treatment.

# Social Information Communications

Figure C.1 is an English language version of how Social Information was displayed to participants during each round.

Other participants reported the following responses regarding contagion:

| **Do you believe that you can contract Hepatitis C from:** | **Person A** | **Person B** | **Person C** |
| --- | --- | --- | --- |
| Sweaty gym equipment? | No | No | No |
| Kissing an infected person? | No | No | No |
| Breastfeeding? | No | No | Yes |
| Unprotected sex with an infected person? | Yes | Unsure | Yes |
| Tattoo equipment used on an infected person? | Yes | Yes | Yes |
| Needle sharing? | Yes | Yes | Yes |

Other participants reported the following responses regarding symptoms:

| **Symptom of Hepatitis C?** | **Person A** | **Person B** | **Person C** |
| --- | --- | --- | --- |
| Rash | Unsure | Unsure | Unsure |
| Headaches | No | No | No |
| Vomiting | Yes | Unsure | No |
| Fever | Unsure | Yes | Yes |
| Fatigue | Unsure | Yes | Yes |
| Loss of Appetite | Yes | Yes | Yes |
| Diarrhea | Unsure | Unsure | Unsure |
| Hair Loss | Unsure | Unsure | No |


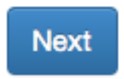


Figure C.1: Social Information (English language version) as seen by participants in the Accurate SI treatment during Round 1 of the experiment.

# Oﬀicial Information Posters

Only German language versions of the posters were shown to participants in the “bots” condition. English language versions are included here for reader convenience.


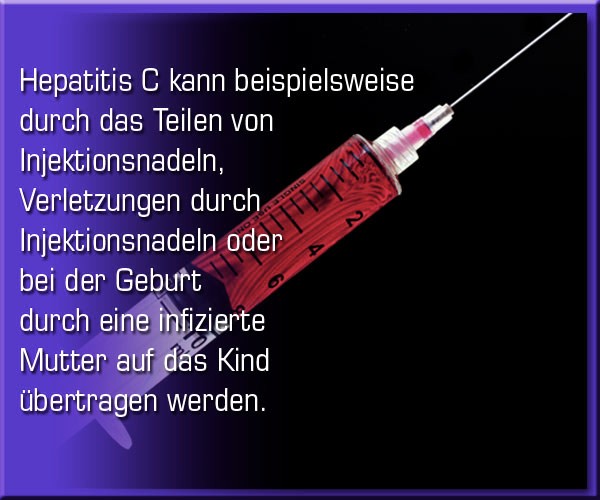

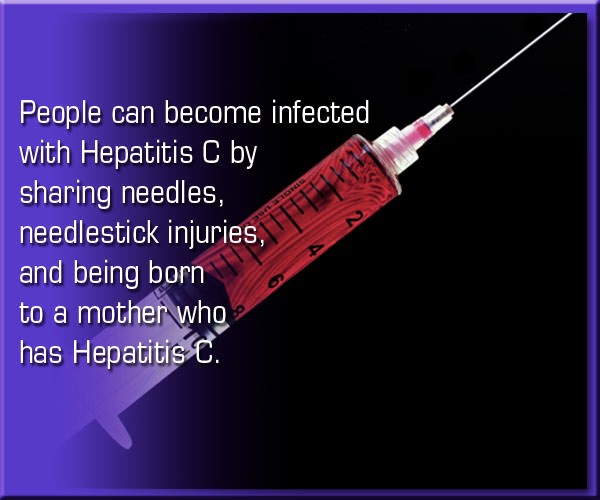


(a) German Language Version (b) English Language Version

Figure D.1: Poster directly relevant to knowledge questions with topics: Needle Sharing.


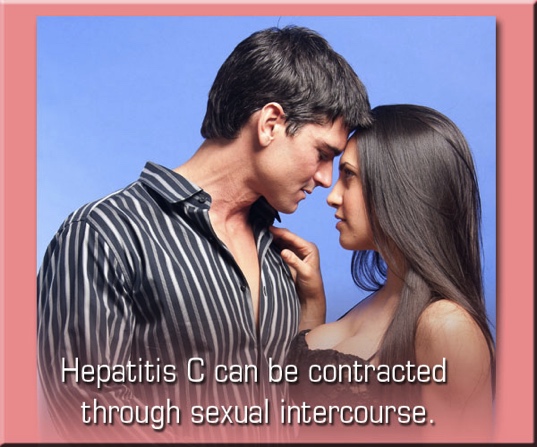

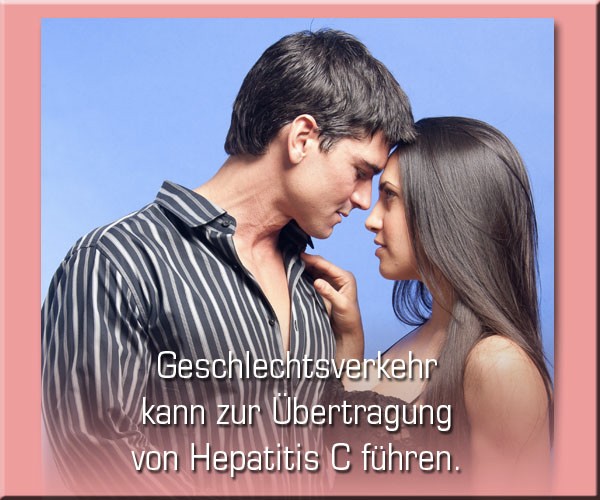


(a) German Language Version (b) English Language Version

Figure D.2: Poster directly relevant to knowledge questions with topics: Unprotected Sex.


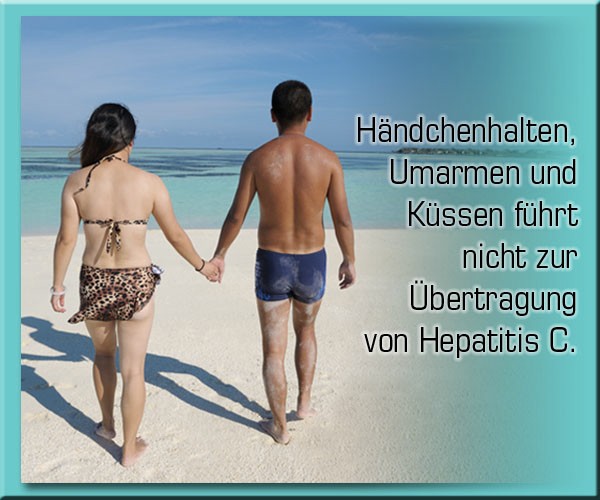

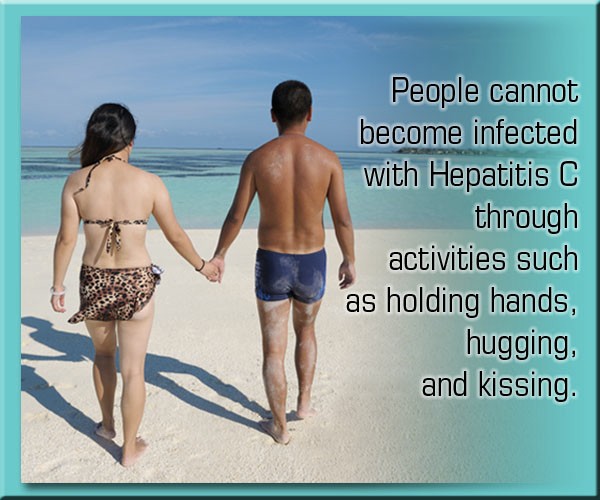


(a) German Language Version (b) English Language Version

Figure D.3: Poster directly relevant to knowledge questions with topics: Kissing.


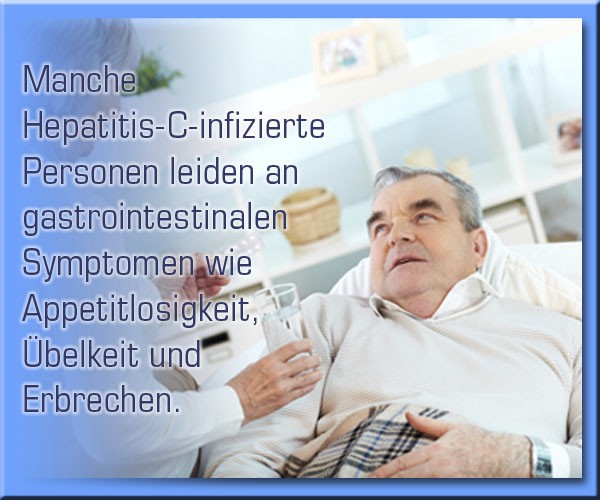

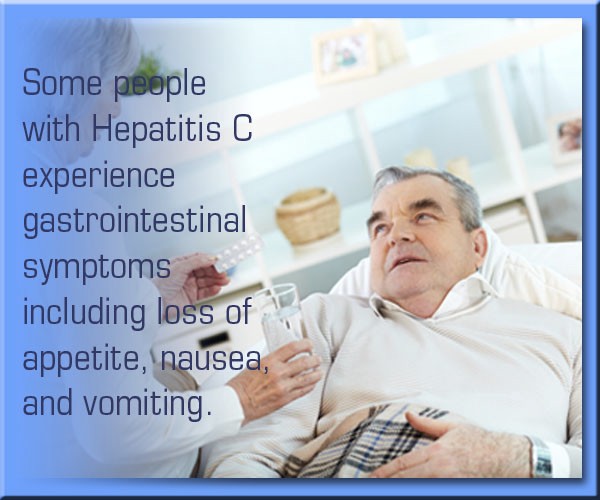


(a) German Language Version (b) English Language Version

Figure D.4: Poster directly relevant to knowledge questions with topics: Vomiting, Loss of Appetite.


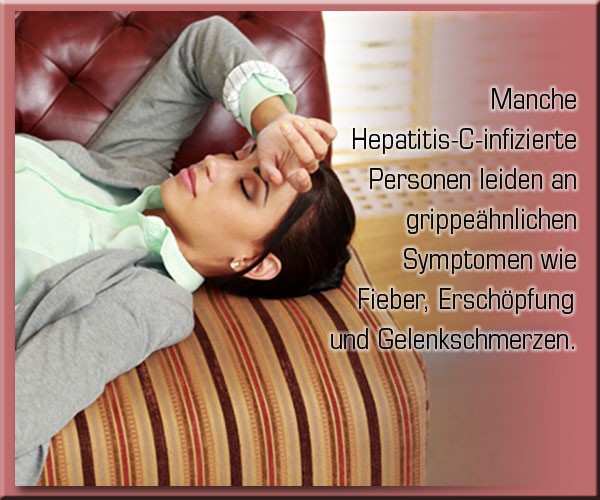

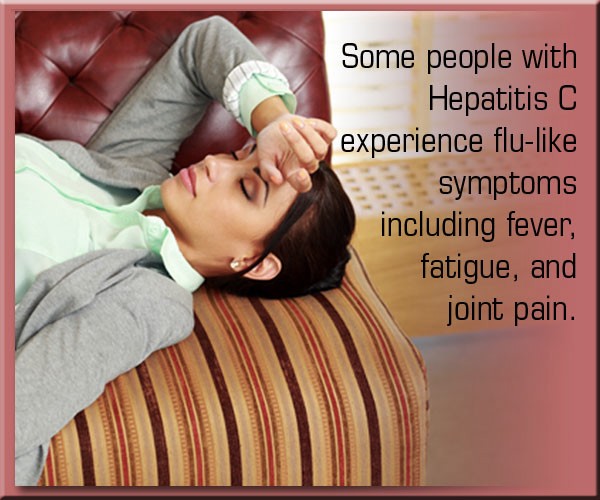


(a) German Language Version (b) English Language Version

Figure D.5: Poster directly relevant to knowledge questions with topics: Fever, Fatigue.


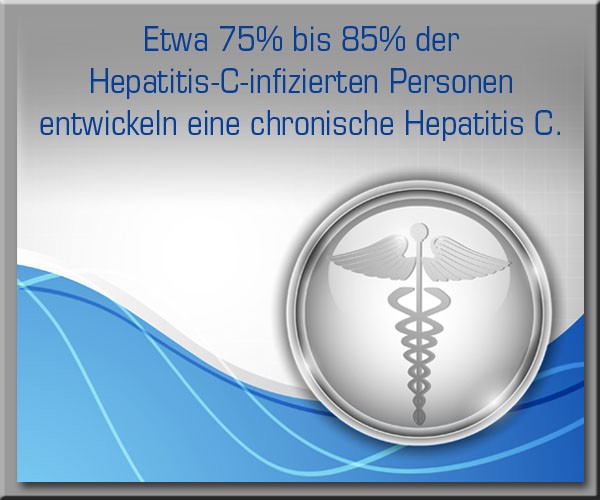

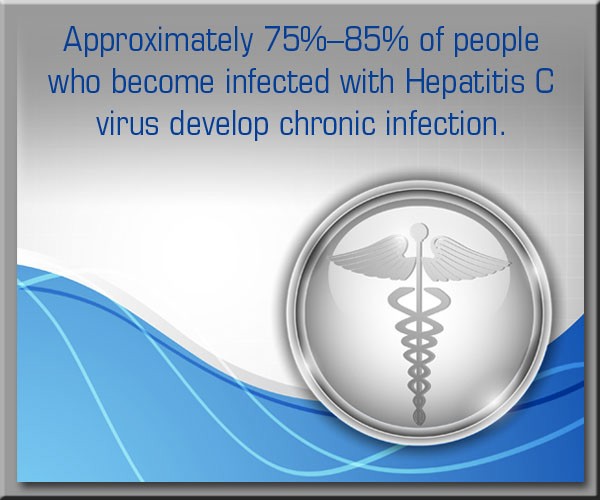


(a) German Language Version (b) English Language Version

Figure D.6: Poster not directly relevant to any knowledge questions.


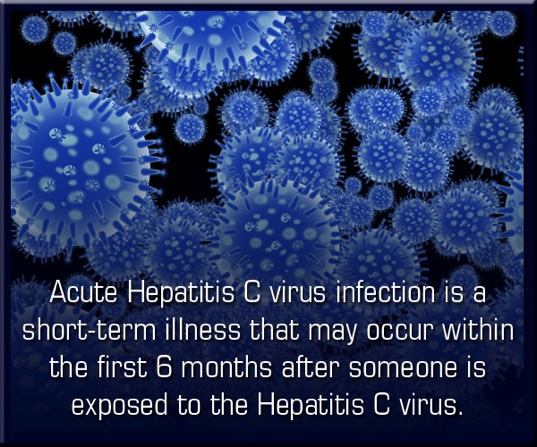

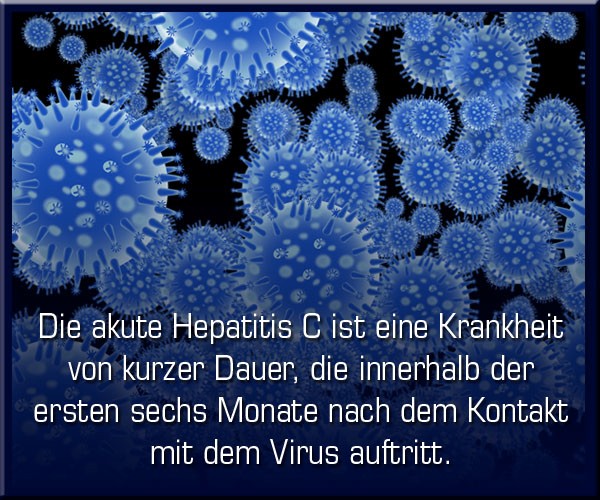


(a) German Language Version (b) English Language Version

Figure D.7: Poster not directly relevant to any knowledge questions.


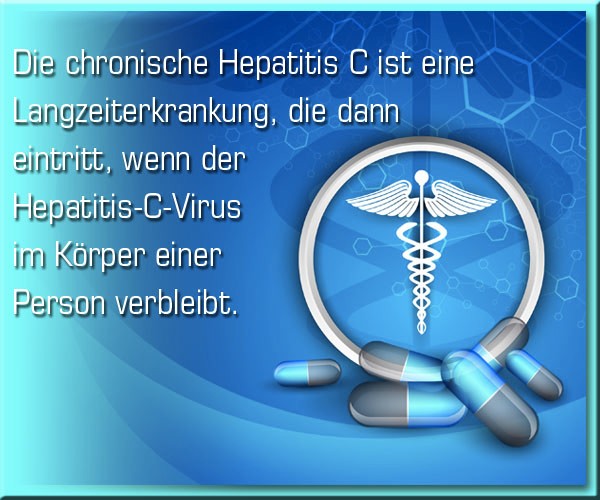

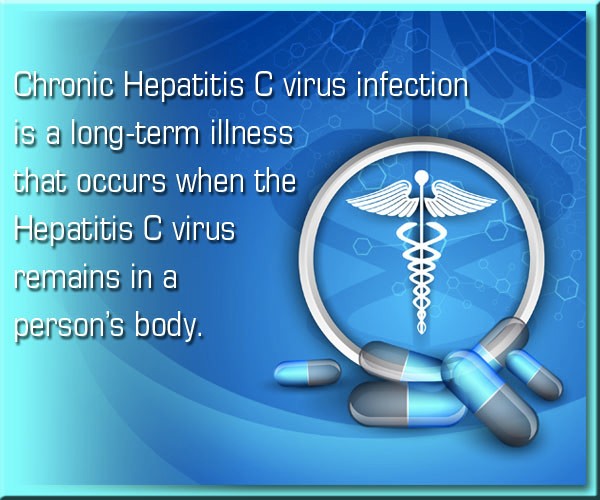


(a) German Language Version (b) English Language Version

Figure D.8: Poster not directly relevant to any knowledge questions.


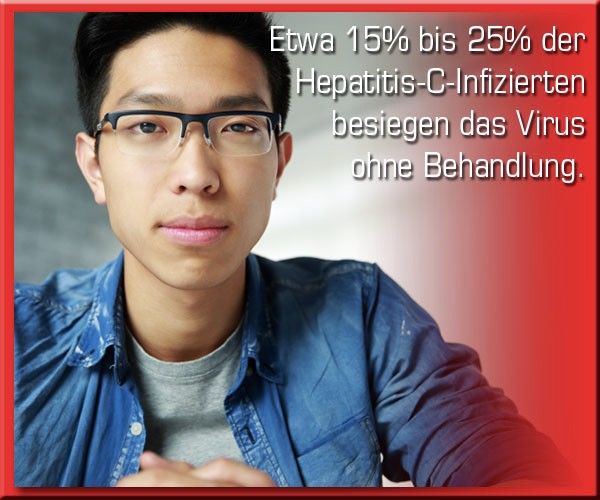

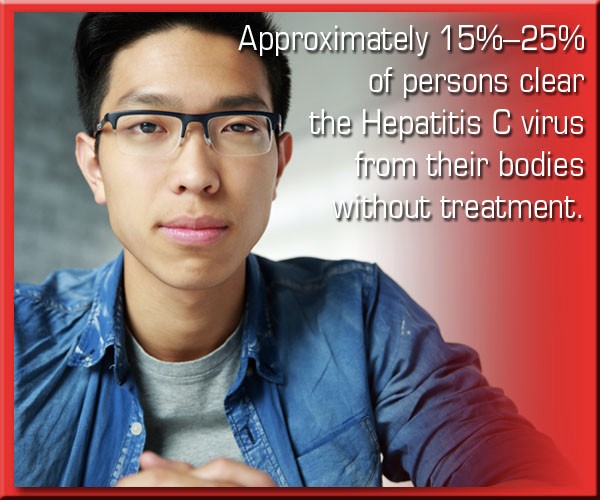


(a) German Language Version (b) English Language Version

Figure D.9: Poster not directly relevant to any knowledge questions.


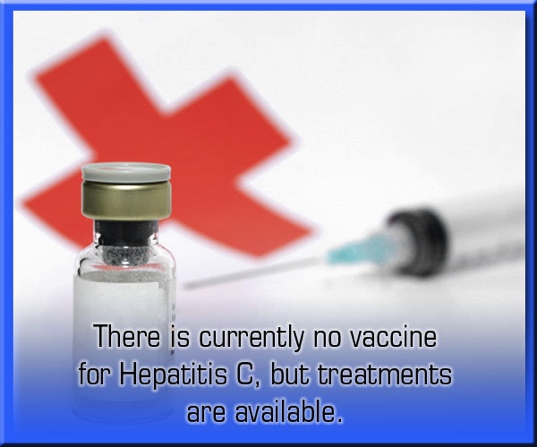

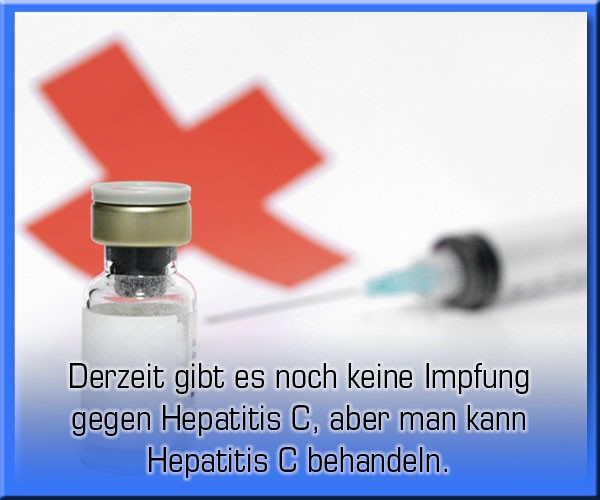


(a) German Language Version (b) English Language Version

Figure D.10: Poster not directly relevant to any knowledge questions.


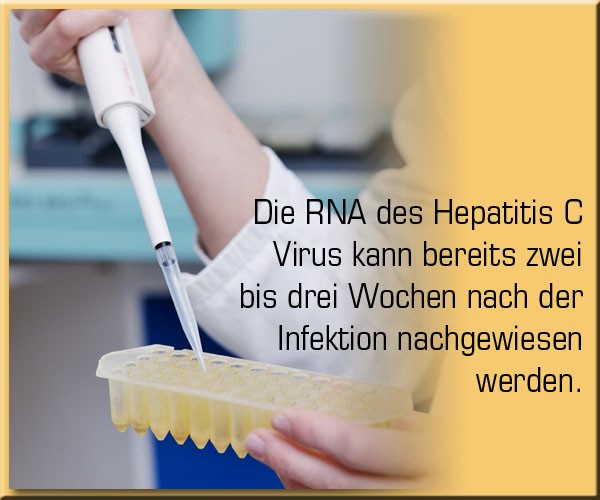

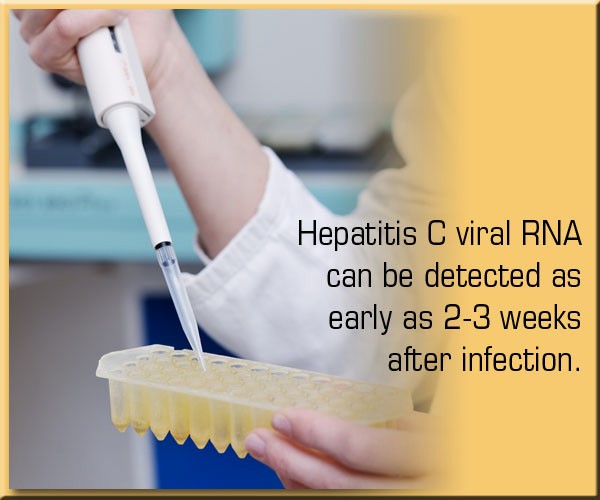


(a) German Language Version (b) English Language Version

Figure D.11: Poster not directly relevant to any knowledge questions.


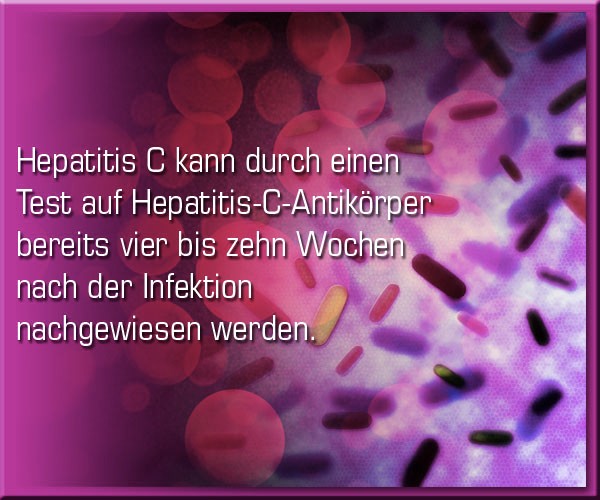

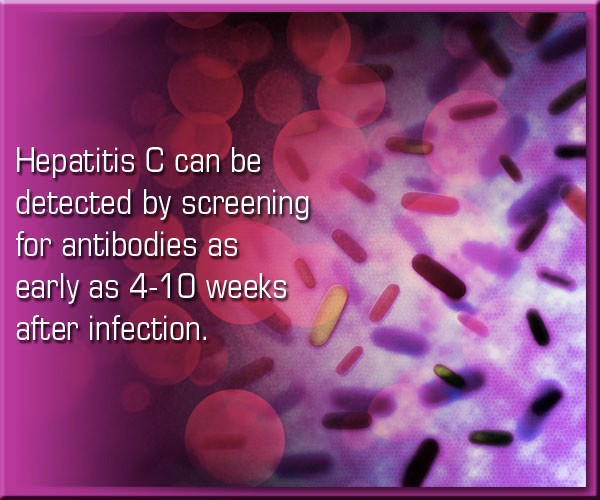


(a) German Language Version (b) English Language Version

Figure D.12: Poster not directly relevant to any knowledge questions.


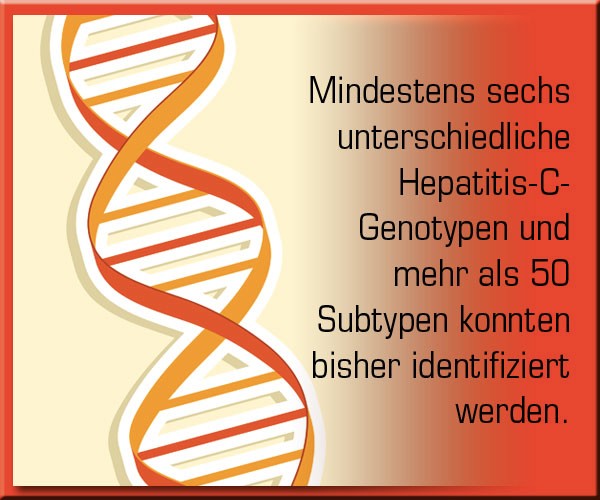

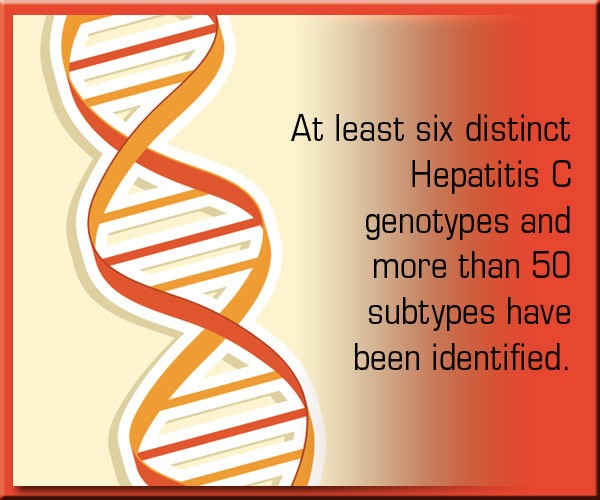


(a) German Language Version (b) English Language Version

Figure D.13: Poster not directly relevant to any knowledge questions.


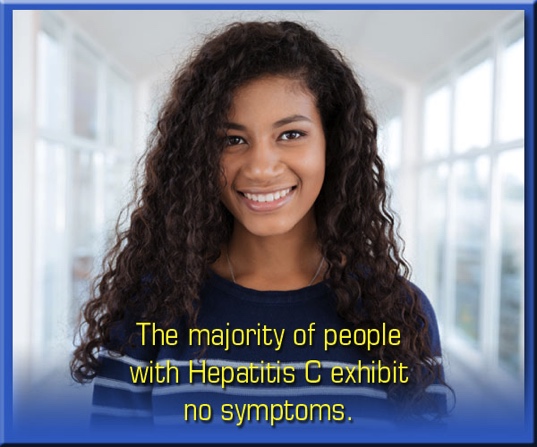

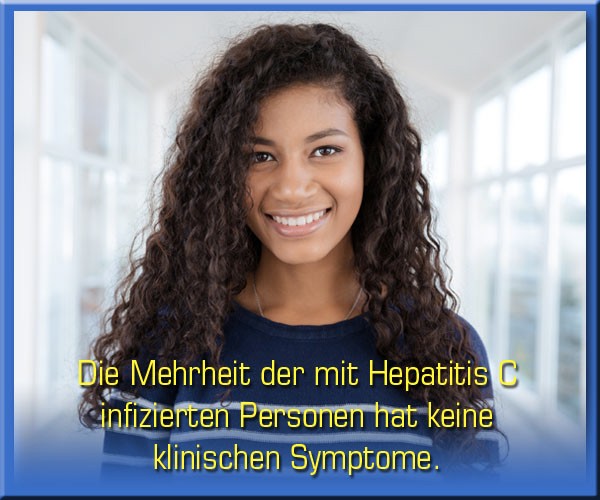


(a) German Language Version (b) English Language Version

Figure D.14: Poster not directly relevant to any knowledge questions.


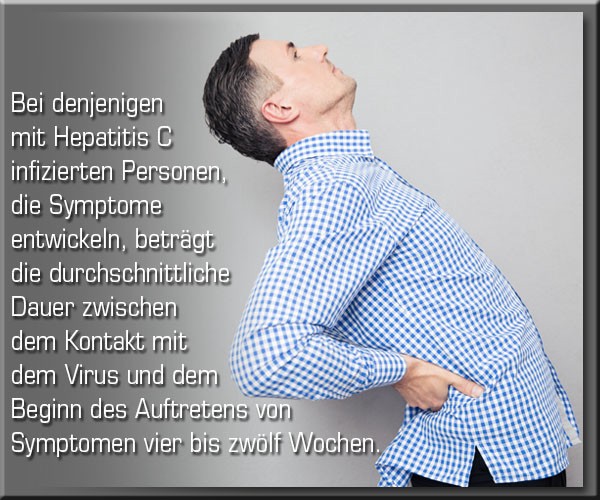

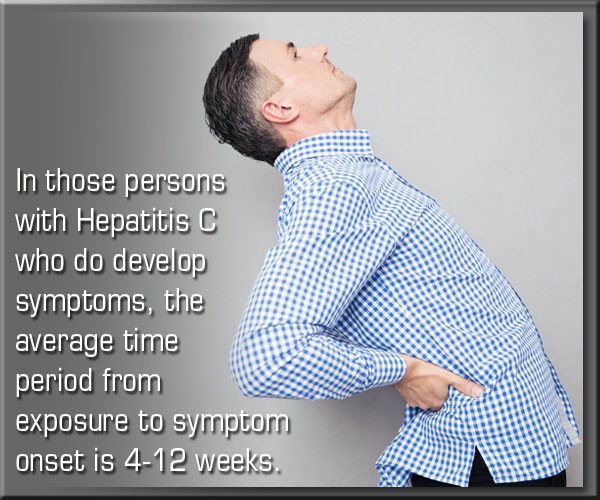


(a) German Language Version (b) English Language Version

Figure D.15: Poster not directly relevant to any knowledge questions.

# E Symptom and Contagion Questions

Below we show the text of the Hepatitis C symptom and contagion questions – exactly as they were displayed to participants during the study.


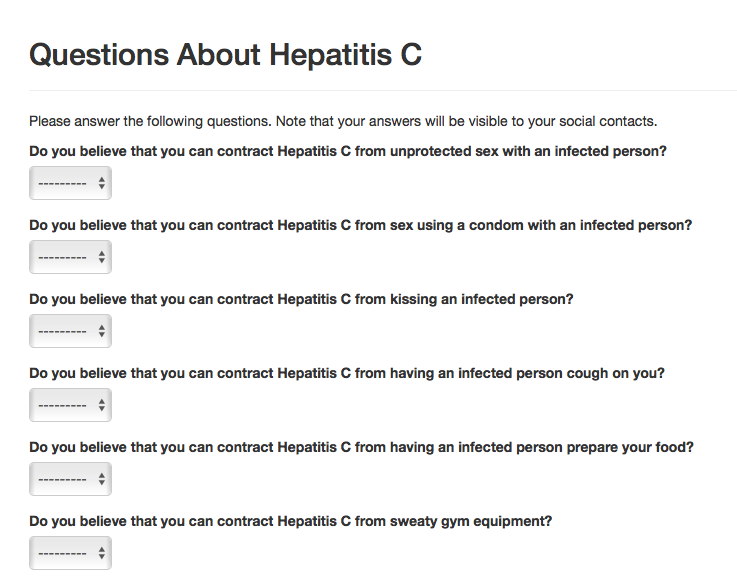


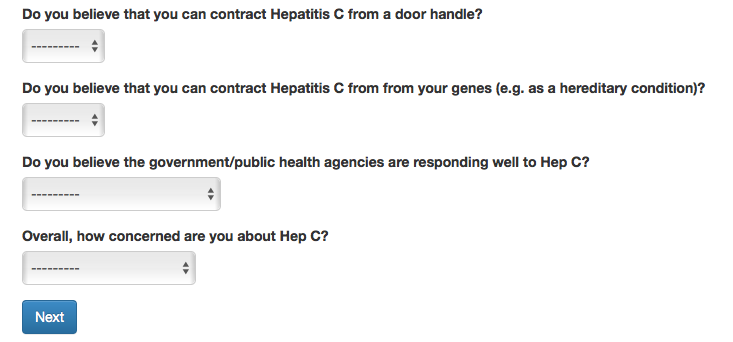


References

R Core Team. (2019). R: A language and environment for statistical computing [Computer software manual].

Vienna, Austria. Retrieved from [https://www.R-project.org/](http://www.R-project.org/)

Viechtbauer, W. (2010). Conducting meta-analyses in R with the metafor package. *Journal of Statistical Software*, *36*(3), 1–48. Retrieved from <http://www.jstatsoft.org/v36/i03/>
